# TMEM189 negatively regulates the stability of ULK1 protein and cell autophagy

**DOI:** 10.1038/s41419-022-04722-y

**Published:** 2022-04-07

**Authors:** Jiahong Yu, Liujing Qu, Yan Xia, Xuan Zhang, Jinqiu Feng, Mengyuan Duan, Pengli guo, Yaxin Lou, Ping Lv, Wenping Lu, Yingyu Chen

**Affiliations:** 1 Department of Immunology, Peking University School of Basic Medical Sciences; NHC Key Laboratory of Medical Immunology, Peking University, 38 Xueyuan Road, Beijing, 100191 China; 2 Department of Clinical Laboratory, The Affiliated Yantai Yuhuangding Hospital of Qingdao University, 20 Yuhuangding East Road, Yantai, Shandong Province 264000 China; 3 Medical and Healthy Analytical Center, Peking University, 38 Xueyuan Road, Beijing, 100191 China; 4 Department of Hepatobiliary Surgery, First Medical Center, Chinese PLA General Hospital, 28 Fuxing Road, Beijing, 100853 China; 5 Center for Human Disease Genomics, Peking University, Beijing, 38 Xueyuan Road, Beijing, 100191 China

**Keywords:** Macroautophagy, Ubiquitylation

## Abstract

ULK1 is crucial for initiating autophagosome formation and its activity is tightly regulated by post-translational modifications and protein-protein interactions. In the present study, we demonstrate that TMEM189 (Transmembrane protein 189), also known as plasmanylethanolamine desaturase 1 (PEDS1), negatively regulates the proteostasis of ULK1 and autophagy activity. In TMEM189-overexpressed cells, the formation of autophagesome is impaired, while *TMEM189* knockdown increases cell autophagy. Further investigation reveals that TMEM189 interacts with and increases the instability of ULK1, as well as decreases its kinase activities. The TMEM189 N-terminal domain is required for the interaction with ULK1. Additionally, TMEM189 overexpression can disrupt the interaction between ULK1 and TRAF6, profoundly impairs K63-linked polyubiquitination of ULK1 and self-association, leading to the decrease of ULK1 stability. Moreover, in vitro and in vivo experiments suggest that *TMEM189* deficiency results in the inhibition of tumorigenicity of gastric cancer. Our findings provide a new insight into the molecular regulation of autophagy and laboratory evidence for investigating the physiological and pathological roles of TMEM189.

## Introduction

Autophagy is a highly conserved intracellular self-digestive and catabolic process that serves as a quality control mechanism. During the initiation of autophagy, the cytoplasmic contents (e.g., lipids, damaged organelles, and long-lived proteins) are enveloped into a double-membrane sac, termed the isolation membrane or phagophore. With its elongation and expansion, the phagophore closes to form a double-membrane autophagosome. The autophagosome then docks and fuses with the late endosome/lysosome to generate the autolysosome, which allows the resident hydrolases to degrade the sequestered cargo to provide the necessary nutrient supply to the cells [1, 2]. In humans, defective autophagy has been closely connected to cancer, neurodegeneration and autoimmune diseases [3].

The process of de novo autophagosome formation has been shown to be regulated by several core machinery complexes, including the ATG1/ULK1 complex, the PIK3C3-BECN1 complex, transmembrane proteins ATG9 cycling system and WIPI complexes, and two ubiquitin-like protein conjugation systems (ATG12–ATG5/ATG16L1 and MAP1ALC3-PE). The ULK1 complex contains serine/threonine ULK1, RB1CC1 (RB inducible coiled-coil 1)/FIP200, ATG13, and ATG101, which is essential for the initiation of autophagy [4]. This complex is highly regulated through post-translational modification and protein-protein interactions. E3 ubiquitin ligase TRAF6 is a ULK1-binding molecule, which can promote ULK1 ubiquitylation by k63-linked chains and its subsequent stabilization, self-association, and function [5]. The molecular chaperone complex, HSP90-Cdc37, and chaperone-like protein C1QBP/p32, can stabilize and activate ULK1 [6, 7]. Ubiquitin-specific protease 20 (USP20) acts as a positive regulator of autophagy initiation through stabilizing ULK1 [8]. Conversely, mitochondrial outer-membrane E3 ligase MUL1 promotes the Lys48-linked ubiquitination of ULK1 and negatively regulates selenite-induced mitophagy [9]. The Cul3-KLHL20 ubiquitin ligase was associated with feedback regulation, promoting the termination of autophagy through the degradation of multiple subunits of the ULK1 and PIK3C3 complexes; this mechanism is important for cell survival and muscle homeostasis [10]. E3 ubiquitin ligase NEDD4L downregulates autophagy and cell growth by mediating ULK1 proteasomal degradation [11].

TMEM189 (Transmembrane protein 189), also known as plasmanylethanolamine desaturase 1 (PEDS1), which is necessary for plasmalogen biosynthesis [12, 13]. It is highly evolutionary conserved and widely expressed in a variety of tissues and organs. In humans, TMEM189/PEDS1 and UBE2V1 are neighboring genes, expressed as either two separate transcripts encoding TMEM189/PEDS1 and UBE2V1 proteins, or as a rare read-through TMEM189/PEDS1-UBE2V1 fusion gene transcript [14]. A very recent study revealed that TMEM189 was strongly correlated with resistance to ferroptosis inducers [15]. It could abrogate fatty acyl-CoA reductase FAR1-induced ferroptosis through plasmalogen synthesis, and suggests TMEM189 as a promising druggable target for anticancer therapy. To date, there have been no reports of the involvement of TMEM189 in autophagy. Here, we for the first time demonstrate that TMEM189 negatively regulates autophagy via downregulating ULK1 signaling. Our findings provide novel insight for revealing the molecular regulation of the autophagic network and provide an experimental basis for investigating the physiological and pathological roles of TMEM189.

## Results

### TMEM189 negatively regulates the formation of autophagosomes

TMEM189 was originally identified as an autophagy-related gene through a high-throughput, cell based functional screening [16]. We designed a series of experiments to further explore the potential relationship between TMEM189 and autophagy. Data from experiments confirmed that overexpression of TMEM189 caused a significant reduction of autophagosome formation, which was monitored by a GFP-LC3B puncta assay and LC3B turnover. As shown in Fig. S1, the number of GFP-LC3B puncta per cell was decreased in TMEM189-overexpressing HeLa cells comparing with control cells, both in the basal or starvation conditions (Fig. S1a, b). Similar findings were observed in rapamycin (RAPA)-treated cells (Fig. S1a, b). Consistent with the results of confocal, overexpression of TMEM189 reduced the levels of endogenous LC3B-II (Fig. S1c, d). Bafilomycin A1 (BafA1) is an inhibitor of the late phase of autophagy, which blocks autophagosomes-lysosomes fusion by inhibiting the vacuolar H^+^ ATPase (V-ATPase). Although BafA1 treatment resulted in an increase of LC3B lipidation in TMEM189- and vector-transfected cells, the number of GFP-LC3B puncta and accumulation of LC3B-II in the former cells was less than that of the latter cells (Fig. S1a–d), indicating that overexpression of TMEM189 might impair the synthesis of autophagosomes. We next analyzed the level of SQSTM1/P62 degradation in HeLa cells. The immunoblot analysis revealed a reduction in the level of SQSTM1 in vector-transfected cells treated by EBSS treatment (Fig. S1e), while TMEM189 overexpression failed to affect the levels of SQSTM1 protein (Fig. S1f), indicating that autophagy flux might be impaired in the presence of TMEM189.

**Fig. 1:**
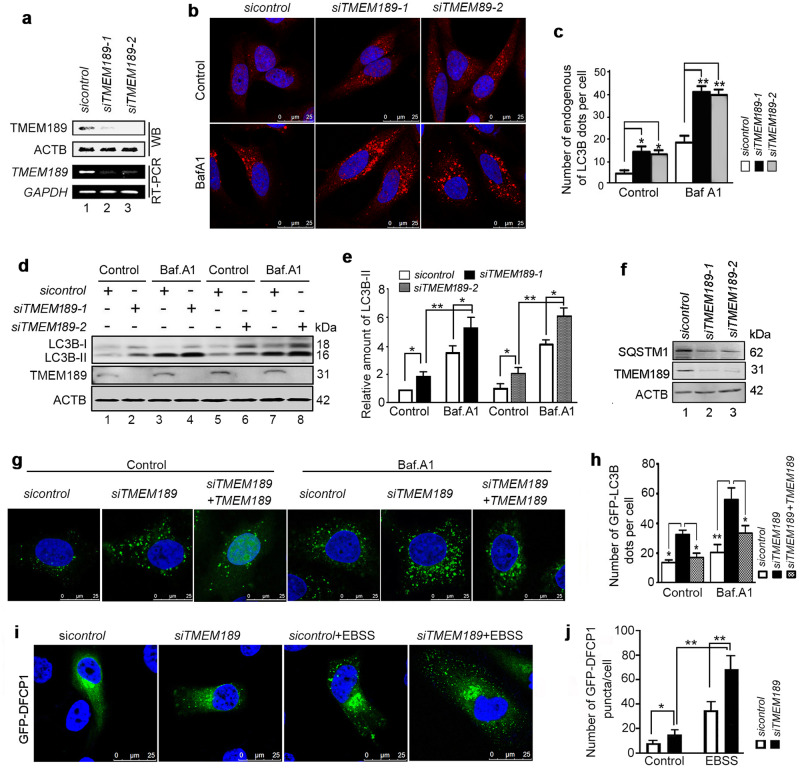
TMEM189 negatively regulates the formation of autophagosome. **a** HeLa cells were transfected with indicated siRNAs for 48 h, the mRNA and protein levels of TMEM189 were detected by RT-PCR and western blotting, respectively. **b** HeLa cells were transfected with indicated siRNAs for 48 h, and treated with or without BafA1 (10 nM) for the last 4 h. The endogenous LC3B dot distribution was analyzed by immunofluorescence and confocal microscopy. **c** Quantification of endogenous LC3B dots per cell was counted. Data are means ± SD of at least 50 cells scored. **d** HeLa cells were treated as (**b**). The levels of endogenous LC3B-II were detected by western blotting. **e** Quantification of amounts of LC3B-II relative to ACTB in cells. Average value in *sicontrol*-transfected cells without BafA_1_ was normalized as 1. Data are means ± SD of results from 3 experiments. **f** HeLa cells were transfected with indicated siRNAs for 48 h, the levels of SQSTM1/P62 were analyzed by western blotting. **g** The stable GFP-LC3B-expressing HeLa cells were transfected with indicated siRNA-1/plasmids for 48 h, then incubated in the presence or absence of BafA1 (10 μM, 4 h). The GFP-LC3B dot distribution was observed by confocal microscopy. **h** Quantification of GFP-LC3B dots per cell was counted. Data are means ± SD of at least 50 cells scored. **i** The GFP-DFCP1-expressing HeLa cells were transfected with indicated siRNAs for 48 h, then incubated with or without EBSS for the last 1 h. The GFP-DFCP1 dot distribution was observed by confocal microscopy. **j** Quantification of GFP-DFCP1 dots per cell was counted. Data are means ± SD of at least 50 cells. *P < 0.05, **P < 0.01.

Further analysis was performed in *TMEM189*-silenced HeLa cells, two *siRN*A against *TMEM189* were demonstrated to be effective using both RT-PCR and Western blot (Fig. 1a). Data from experiments proved that *TMEM189* knockdown increased the number of endogenous LC3B dots and the accumulation of endogenous LC3B-II comparing to that control cells (Fig. 1b–e), while BafA1 treatment further elevated the lipidation of LC3B. Simultaneously, the levels of SQSTM1 were decreased in *TMEM189*-silenced cells (Fig. 1f), suggesting that the depletion of *TMEM189* promotes functional autophagy. We next performed a rescue experiment by co-transfecting the TMEM189 plasmid with *siTMEM189-1*. As shown in Fig. 1g and h, the increased GFP-LC3B puncta induced by si*TMEM189-1* was downregulated when combined with the *TMEM189* plasmid with or without BafA1. These findings suggest that the TMEM189 is a negative regulator of autophagy.

**Fig. 2:**
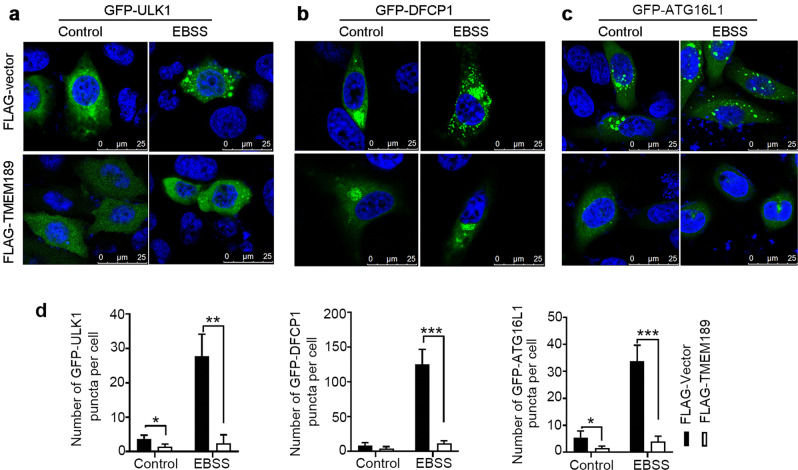
Overexpression of TMEM189 inhibits the formation of autophagosome. HeLa cells were cotransfected with indicated plasmids for 24 h, then incubated with EBSS for 1 h. The dot distribution of GFP-ULK1 (**a**), GFP-DFCP1 (**b**) and GFP-ATG16L1 (**c**) was observed by confocal microscopy. **d** Quantification of puncta per cell was counted. Data are means ± SD of at least 50 cells. *P < 0.05, **P < 0.01, ***P < 0.001.

To determine which step of autophagy is regulated by TMEM189, we examined reporters for autophagic structures at different stages. The autophagosome formation sites on the ER are labeled by the ULK1 complex. The PtdIns(3)P-enriched omegasomes are marked by ZFYVE1/DFCP1, while the ATG16L1 labels IMs but not closed autophagosomes [17]. LC3 labels IMs, autophagosomes, amphisomes and autolysosomes. As shown in Fig. 2, comparison with vector-transfected HeLa cells, TMEM189 overexpression (Fig. S2) significantly decreased the puncta of ULK1, DFCP1 and ATG16L1 under amino acid starvation. Meanwhile, the fluorescent signals of these proteins has also weakened in TMEM189-overexpressed cells. Conversely, knockdown of *TMEM189* increased the dot distribution of DFCP1 in both basal and EBSS-induced HeLa cells (Fig. 1i, j). These results suggest that TMEM189 acts on the upstream of autophagy and negatively regulates the formation of autophagosome.

### TMEM189 interacts with ULK1 via its N-terminal

**Fig. 3:**
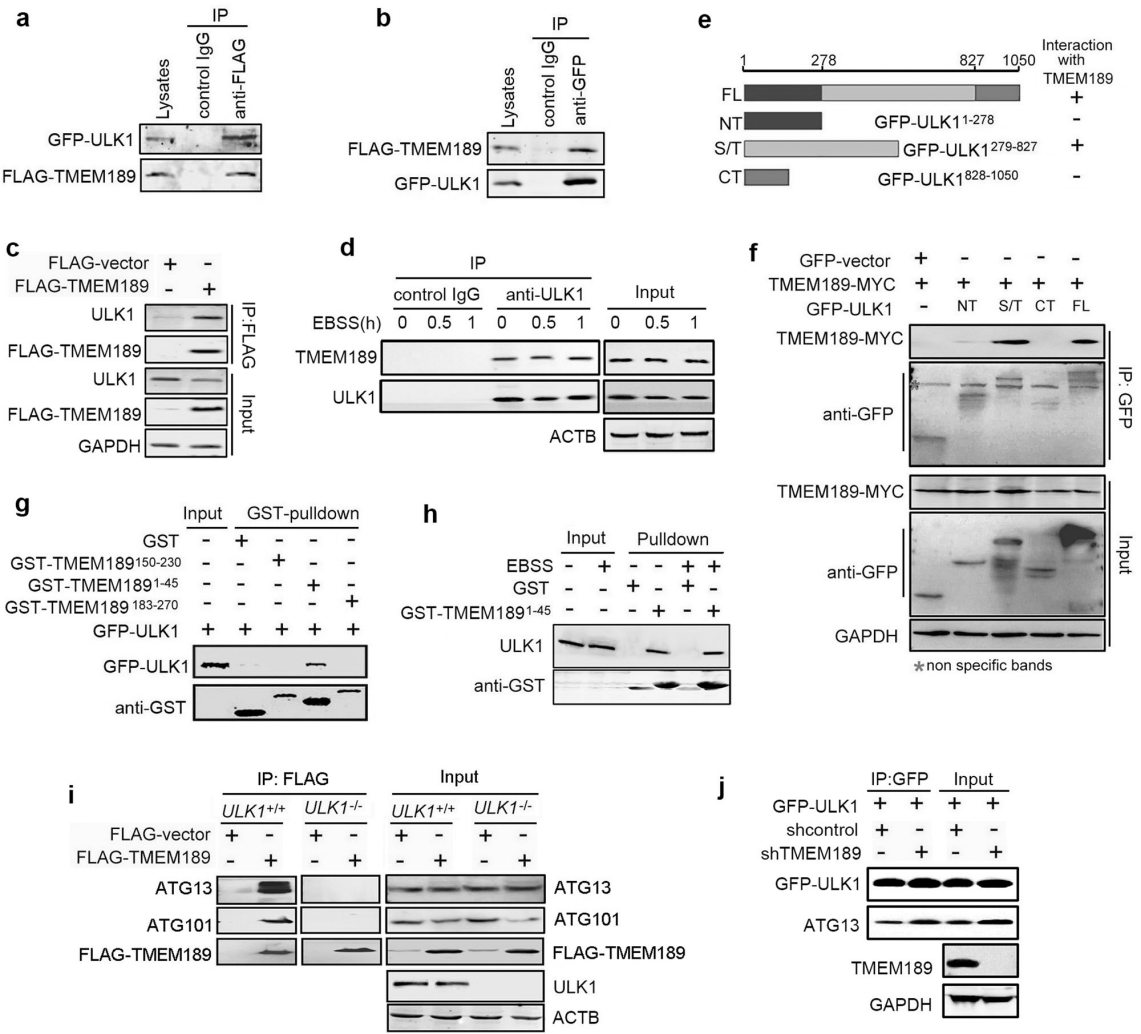
TMEM189 interacts with ULK1 via its N-terminal and knockdown of *TMEM189* increases ULK1-ATG13 interaction. **a**, **b** HEK293T cells were cotransfected with GFP-ULK1 and FLAG-TMEM189 plasmids for 24 h, then cell lysates were subjected to IP using an anti-FLAG, an anti-GFP or a control IgG as indicated. GFP-ULK1 and FLAG-TMEM189 proteins were detected in the immunoprecipitates by western blotting. **c** HEK293T cells were transfected with indicated plasmids for 24 h, then cell lysates were subjected to IP using an anti-FLAG. Endogenous ULK1 proteins were detected in the immunoprecipitates by western blotting. **d** HeLa cells were incubated with or without EBSS for indicated time, then cell lysates were subjected to IP using an anti-ULK1 or a control IgG. The endogenous ULK1 and TMEM189 proteins were detected in the immunoprecipitates by western blotting. **e** Construction of truncated GFP-ULK1 plasmids. **f** HEK293T cells were cotransfected with indicated plasmids for 24 h, then cell lysates were subjected to IP using an anti-GFP. GFP-ULK1 and TMEM189-MYC proteins were detected in the immunoprecipitates by western blotting. **g** GST-TMEM189^1-45^, GST-TMEM189^150-230^, GST-TMEM189^183-270^ fusion protein and the GST protein were purified and immobilized on Glutathione-Sepharose beads, then incubated with HEK293T cell lysates containing GFP-ULK1. Proteins retained on Glutathione-Sepharose were then blotted using the indicated antibodies. **h** Purified GST-TMEM189^1-45^ and GST protein were incubated with HEK293T cell lysates with or without EBSS incubation for 30 min, the endogenous ULK1 and GST-fusion protein were detected in the washed beads by western blotting. **i** *ULK1* wild-type (*ULK1*^*+/+*^) and *ULK1* knockout (*ULK1*^*−/−*^) HeLa cells were transfected with FLAG-vector or FLAG-TMEM189 for 24 h, then cell lysates were subjected to IP using an anti-FLAG. Endogenous ATG13, ATG101 and FLAG-TMEM189 proteins were detected in the immunoprecipitates by western blotting. **j** The stable *shcontrol*- or *shTMEM189*-expressing HeLa cells were transfected with GFP-ULK1 plasmids. Cell lysates were immunoprecipitated using an anti-GFP and analyzed by immunoblotting with anti-ATG13 antibody. Simultaneously, 10% cell lysates were used to immunoblotting.

To investigate the molecular mechanism, we began to explore the potential interaction between TMEM189 and several autophagy-related molecules. Data from a co-immunoprecipitation (CO-IP) assay demonstrated that association of FLAG-TMEM189 with GFP-ULK1 and endogenous ULK1 was detected in HEK293T cells by reciprocal immunoprecipitations (Fig. 3a–c). The interaction of the endogenous ULK1 and TMEM189 was also observed with or without EBSS treatment (Fig. 3d).

The ULK1 protein is composed of 1050 amino acids, of which the N-terminal (amino acids 1–278, NT) exhibits protein kinase activity, 279–827 amino acid residues are rich in serine/threonine (S/T) domains, and 828–1050 amino acids have a C-terminal (CT) domain. Therefore, we constructed several truncation of GFP-ULK1 according to its functional domains (Fig. 3e) and investigated which ULK1 domain was responsible for the TMEM189 interaction. Data from CO-IP assay suggest that the S/T domain of ULK1 is sufficient to interact with TMEM189 (Fig. 3f), whereas the CT domain, which binds ATG13 [18], shows no interaction. Further experiments indicate that the N-terminal (amino acids 1–45) of TMEM189 was responsible for the interaction with GFP-ULK1 (Fig. 3g) and endogenous ULK1 (Fig. 3h). These data collectively suggest that ULK1 is a physiological and direct binding protein of TMEM189.

Next we checked whether TMEM189 associates with other ULK1-interacting proteins. As shown in Fig. 3i, FLAG-TMEM189 could bind endogenous ATG13 and ATG101 in *ULK1*^*+/+*^ HeLa cells (Fig. 3i, left panel). However, both TMEM189-ATG13 and TMEM189-ATG101 interaction were completely abrogated in *ULK1* knockout (*ULK1*^*−/−*^) cells (Fig. 3i, right panel), suggesting that TMEM189 may indirectly bind ATG13 and ATG101.

### Knockdown of *TMEM189* increases the stability of ULK1 and its kinase activity

**Fig. 4:**
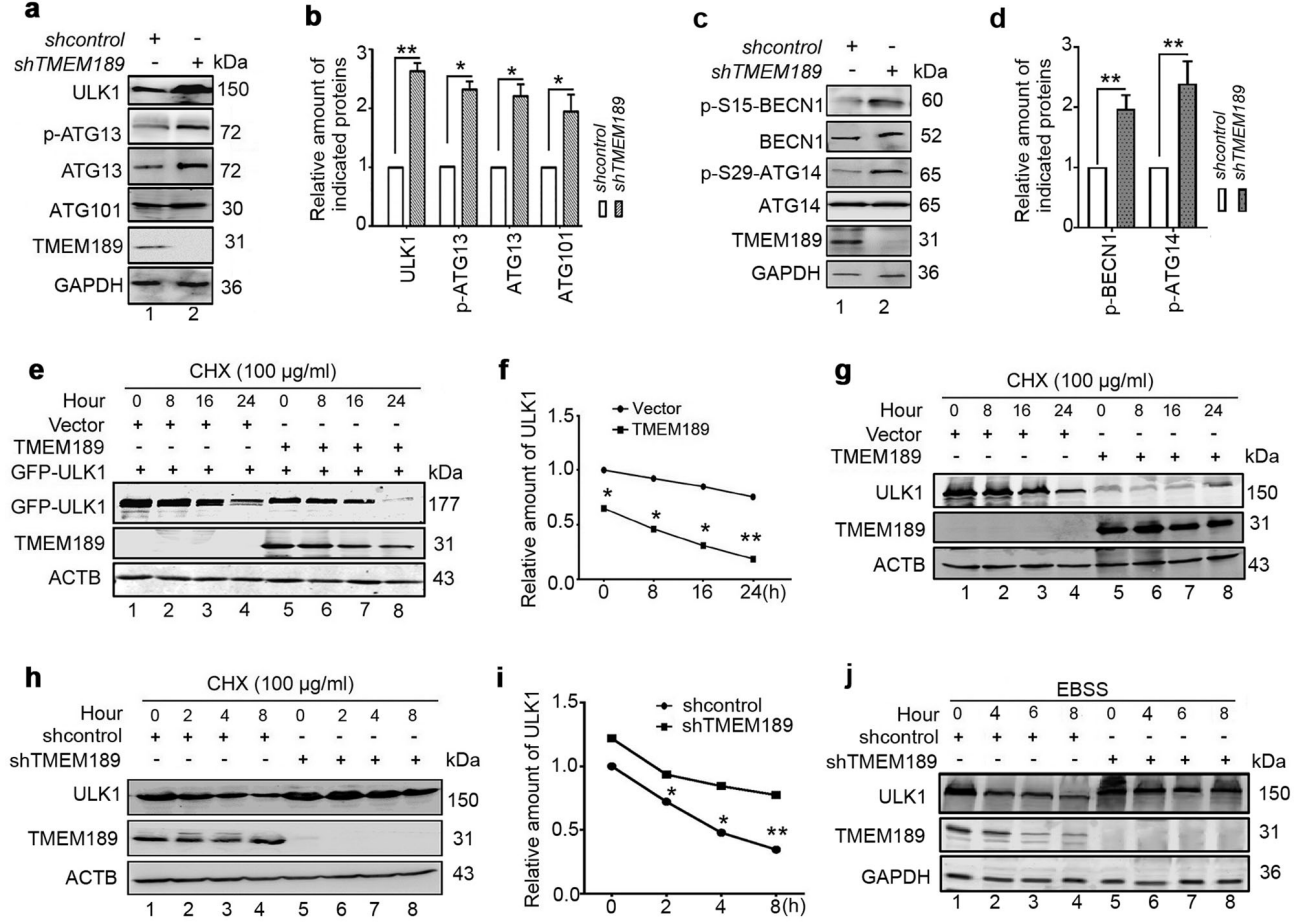
*TMEM189* knockdown increases ULK1 stability and kinase activity. **a**, **c** Western blot analysis of endogenous indicated protein in cell lysates extracted from the stable *shcontrol*- or *shTMEM189*-expressing HEK293T cells. **b**, **d** Quantification of amounts of indicated protein relative to GAPDH in cells. Average value in *shcontro*l-infected cells was normalized as 1. Data are means ± SD of results from 3 experiments. **e** HEK293T cells were cotransfected with indicated plasmids for 24 h, then incubated with cycloheximide (CHX, 100 μg/mL) for indicated time. The levels of GFP-ULK1 and TMEM189 were detected by western blotting. **f** Quantification of GFP-ULK1 proteins (ratio to ACTB) in cells treated as in (**e**). **g** HEK293T cells were transfected with indicated plasmids for 24 h, incubated in cycloheximide (CHX, 100 μg/mL) for indicated time. The levels of endogenous ULK1 were detected by western blotting. **h** The stable *shcontrol*- or *shTMEM189-*expressing HeLa cells were incubated with CHX (100 μg/mL) for indicated time. The levels of endogenous ULK1 were detected by western blotting. **i** Quantification of endogenous ULK1 proteins (ratio to ACTB) in cells treated as in (**h**). **J** The stable *shcontrol*- or *shTMEM189*-expressing HEK293T cells were incubated with EBSS for indicated time. The levels of endogenous ULK1, TMEM189 and GAPDH were detected by western blotting.

In our repeated experiments, we found that the ULK1 protein was reduced in TMEM189-overexpressing cells. Moreover, this phenomenon directly affected the success of the CO-IP assays. Thus, we speculate that TMEM189 may mediate ULK1 proteostasis. Indeed, overexpression of TMEM189 significantly decreased the levels of GFP-ULK1 (Fig. S3a). Conversely, silencing of *TMEM189* with lentiviral-based shRNA led to the upregulation of endogenous ULK1 expression (Fig. 4a, b). Notably, the levels of *ULK1* mRNA were not altered in TMEM189-overexpressing cells (data not shown). We then tested whether ULK1 reduction in TMEM189-overexpressed cells could be attributed to increased proteasomal or lysosomal degradation. As shown in Figs. S3b and 3c, the decreased ULK1 was slightly recovered in MG132-treated cells (Figs. S3b and 3c, lane 4 vs. lane 2). While treatment with Baf.A1 or cotreatment with lysosomal protease inhibitors (E64d+PepA) largely restored the ULK1 expression in TMEM189-overexpressed cells (Figs. S3b and 3c, lane 6 and 8 vs. lane 2), suggesting that overexpression of TMEM189 mainly leads to increased lysosomal degradation of ULK1. Simultaneously, we observed that the levels of endogenous ATG13, phosphorylated ATG13 at S318, ATG101, phosphorylated ATG14L at Ser29 and Beclin-1 at Ser15 were also increased in *TMEM189*-ablated cells (Fig. 4a–d)). In the presence of TMEM189, the levels of ATG13 phosphorylation, ATG13 and ATG101 were markedly decreased (Fig. S3d, e), indicating that TMEM189 maybe antagonistic to the maintenance of ULK1 homeostasisa and kinase activity.

We next examined the formation of the ULK1 complex in *TMEM189* knockdown cells. Compared with control cells, levels of endogenous ATG13 coprecipitated by GFP-ULK1 were increased (Fig. 3j). Levels of endogenous ATG101 or FIP200 coprecipitated by GFP-ULK1, and ATG13 coprecipitated by GFP-ATG101 remained unaltered (Fig. S3f–h). These findings hint that TMEM189 mainly downregulates ULK1-ATG13 interaction and their stability.

Next we analyzed the half-life of ULK1 protein using the protein translation inhibitor, cycloheximide (CHX, 100 μg/mL). As shown in Fig. 4e–g, overexpression of TMEM189 promoted the decay of the GFP-ULK1 and endogenous ULK1 protein. On the contrary, knockdown of *TMEM189* increased the stability of ULK1 protein (Fig. 4h, i). We thus determined the effect of TMEM189 on autophagic dynamics. In control cells, the levels of ULK1 protein were declined after EBSS treatment. In the absence of TMEM189, the degradation of ULK1 protein in HEK293 cells was delayed during the autophagy period (Fig. 4j). Similar results were also observed in CCCP-treated cells (Fig. S4), These data reveal that TMEM189 is a negative regulator of ULK1 stabilization in autophagy process.

### TMEM189 disrupts the interaction between ULK1 and TRAF6, impairs K63-linked polyubiquitination of ULK1

**Fig. 5:**
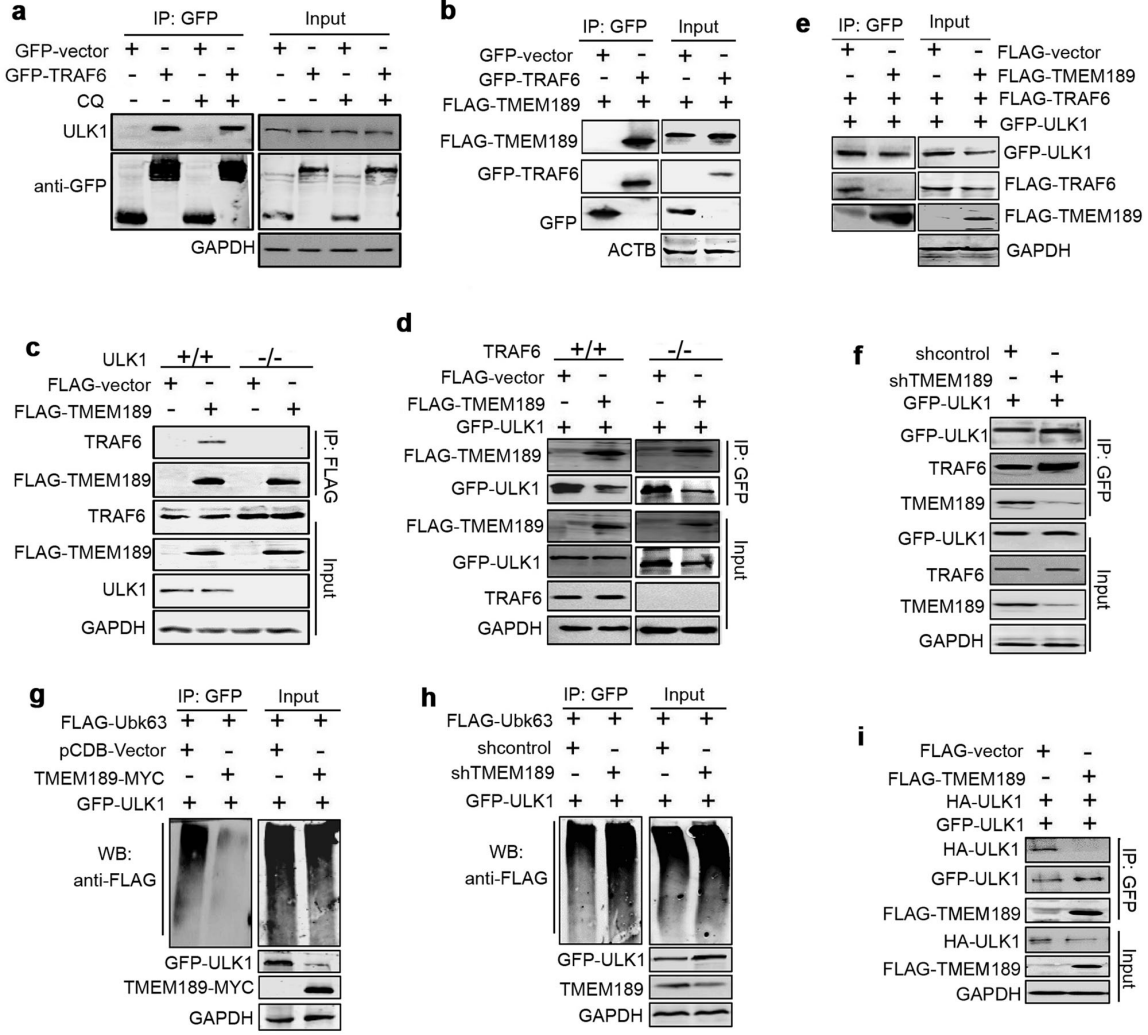
TMEM189 disrupts the interaction between ULK1 and TRAF6, impaires K63-linked ULK1 polyubiquitination. **a** HEK293T cells were transfected with indicated plasmids for 24 h, then treated with or without 50 μM of CQ for 4 h. Cell lysates were subjected to IP using an anti-GFP. The endogenous ULK1 and GFP-TRAF6 proteins were detected in the immunoprecipitates by western blotting. **b** HEK293T cells were cotransfected with indicated plasmids for 24 h, cell lysates were subjected to IP using an anti-GFP. The FLAG-TMEM189 and GFP-TRAF6 proteins were detected in the immunoprecipitates by western blotting. **c** *ULK1*^*+/+*^ and *ULK1*^*−/−*^ HeLa cells were transfected with FLAG-vector or FLAG-TMEM189 for 24 h, then cell lysates were subjected to IP using an anti-FLAG. Endogenous TRAF6 and FLAG-TMEM189 proteins were detected in the immunoprecipitates by western blotting. **d** *TRAF6*^*+/+*^ and *TRAF6*^*−/−*^ HEK293T cells were transfected with indicated plasmids for 24 h, then cell lysates were subjected to IP using an anti-GFP. FLAG-TMEM189 and GFP-ULK1 proteins were detected in the immunoprecipitates by western blotting. **e** HEK293T cells were transfected with indicated plasmids for 24 h, then cell lysates were subjected to IP using an anti-GFP. FLAG-TMEM189, FLAG-TRAF6 and GFP-ULK1 proteins were detected in the immunoprecipitates by western blotting. **f** The stable shcontrol- or shRNF11*5*-expressing HEK293T cells were cotransfected with indicated plasmids for 24 h, then cell lysates were subjected to IP using an anti-GFP. FLAG-TRAF6 and GFP-ULK1 proteins were detected in the immunoprecipitates by western blotting. **g** The cotransfection of HEK293T cells were shown in the figure. After 24 h, these cells were treated with MG132 for 6 h, then the cell lysates were immunoprecipitated with an anti-GFP, and the western blotting was probed with an anti-FLAG antibody to detect ubiquitinated GFP-ULK1 (left panel). Simultaneously, 10% cell lysates were used to western blotting (right panel). **h** The stable shcontrol- or shRNF11*5*-expressing HEK293T cells were cotransfected with indicated plasmids for 24 h, then treated with MG132 for 6 h. The cell lysates were immunoprecipitated with an anti-GFP, and the western blotting was probed with an anti-FLAG antibody to detect ubiquitinated GFP-ULK1. **i** HEK293T cells were transfected with indicated plasmids for 24 h, then cell lysates were subjected to IP using an anti-GFP. HA-ULK1, GFP-ULK1 and FLAG-TMEM189 proteins were detected in the immunoprecipitates by western blotting. Simultaneously, 10% cell lysates were used to western blotting.

Post-translational modifications (e.g., K63-linked ubiquitylation) often regulate the stability of ULK1. In particular, ULK1 is a K63-linked polyubiquitylated protein mediated by TRAF6 E3 ligase [5]. Data from CO-IP assays proved that TRAF6 interacts with ULK1 with or without CQ treatment (Fig. 5a). TMEM189 was also responsible for the binding with GFP-TRAF6 (Fig. 5b) and endogenous TRAF6 (Fig. 5c) in the presence of ULK1. However, the interaction of TMEM189-TRAF6 was disappeared in *ULK1*^−/−^ HeLa cells (Fig. 5c and Fig. S5), suggesting that the association of TMEM189 with TRAF6 depends on the presence of ULK1. On the contrary, *TRAF6* knockout did not hinder the binding of TMEM189 to ULK1 protein (Fig. 5d). Moreover, the association of ULK1 and TRAF6 was defective in TMEM189-overexpressed cells compared with control cells (Fig. 5e). Furthermore, the binding between ULK1 and TRAF6 was enhanced in *TMEM189*-knockdown cells (Fig. 5f), indicating that TMEM189 overexpression may disrupt the interaction of ULK1 with TRAF6 protein.

We next tested whether TMEM189 could affect the K63-linked polyubiquitylation of ULK1 mediated by TRAF6. The results suggested that TMEM189 overexpression decreased K63-linked polyubiquitin in ULK1 immunoprecipitates (Fig. 5g), which was accompanied by a decrease in the levels of ULK1 protein. In contrast, *TMEM189* knockdown increased the K63-linked polyubiquitin of ULK1 (Fig. 5h). We also detected whether TMEM189 influence the ubiquitin ligase activity of TRAF6. As shown in Fig. S6, TMEM189 overexpression failed to affect TRAF6-linked K63 polyubiquitylation compared with that control cells. Collectively, these studies suggest that TMEM189 may disassociate ULK1-TRAF6, prevent K63-linked polyubiquitination of ULK1 and stability.

As ULK1 K63-linked ubiquitylation is required for ULK1 self-association and function [5], we checked for this process in our system. We found that ULK1 was able to self-associate in HeLa cells and TMEM189 overexpression reduced the amount of GFP-ULK interacting with HA-ULK1 (Fig. 5i). On the contrary, *TMEM189* knockdown increased the amount of GFP-ULK binding to HA-ULK1 (Fig. S7), indicating that TMEM189 is resistant for ULK1 self-association.

### Deletion of TMEM189 inhibits the growth of BGC823 cells

By conducting a search of the Kaplan-Meier Plotter online database, (http://kmplot.com/analysis/index.php?p=service&cancer=gastric) [19], we analyzed the correlation between the levels of *TMEM189* mRNA and survival time in 631 gastric cancer patients. As shown in Fig. S8a, compared with patients exhibiting low levels of *TMEM189* mRNA, those with high levels of *TMEM189* mRNA displayed decreased overall survival. Similar prognostic correlation was observed in gender (male and female) and Lauren classification (instestinal and diffuse) (Fig. S8b–e), indicating that the decreased expression of TMEM189 may be a favorable prognostic indicator for patients with gastric cancer.

**Fig. 6:**
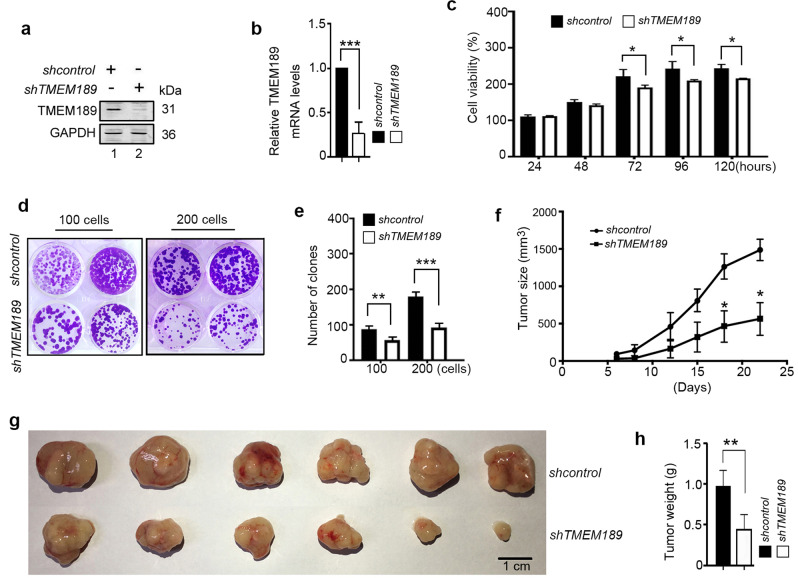
Deletion of *TMEM189* inhibits the growth of BGC823 cells. **a**, **b** The levels of TMEM189 were detected by western blotting and qRT-PCR in stable *shcontrol*- or *shRNF115*-expressing BGC823 cells. **c** The stable *shcontrol*- or *shRNF115*-expressing BGC823 cells were seeded in 96-well plates (3 × 10^3^ cells/well; five replicates), serum starved for 18 h and then pulsed with 10% FBS for indicated time. Cell viability was detected. Data are means ± SD of results from three independent experiments. **d** Representative images of colony formation by indicated cells. **e** Number of clones counted in three independent experiments. **f** The stable *shcontrol*- or *shRNF115*-expressing BGC823 cells were injected subcutaneously in BALB/c nude mice (4 × 10^6^ cells/mouse, *n* = 6), the tumor growth curves were presented in figure. **g**, **h** At 22 days after inoculation, the mice were killed, and the excised xenograft tumors were imaged and weighed. *P < 0.05, **P < 0.01, ***P < 0.001.

To further explore the role of TMEM189 in the development and progression of gastric cancer, we constructed a lentivirus vector *pLVX-shTMEM189* (*shTMEM189*) and screened TMEM189 stably knockdown BGC823 cell line (Fig. 6a). Cell viability and colony formation assays demonstrated that knockdown of *TMEM189* could arrest the growth of BGC823 cells (Fig. 6b–d).

The in vivo effects of TMEM189 were evaluated using a gastric cancer xenograft model established in BALB/C nude mice. These mice were subcutaneously injected with *shcontro*l/BGC823 cells or *shTMEM18*9/BGC823 cells. The tumor growth curves were presented in Fig. 6e, indicating that the *shcontrol* group developed larger tumors than that of the *shTMEM189* group at the site of injection within 22 days. These mice were sacrificed at 22 days after inoculation and tumors from each mouse were photographed. As shown in Fig. 6f and g, the tumor size in the *shTMEM189* group was smaller than that in the *shcontrol* group, suggesting that knockdown of *TMEM189* in BGC823 cells results in the inhibition of tumorigenicity.

## Discussion

In the present study, we identified TMEM189 /PEDS1to be a novel binding partner of ULK1, which functions as a negative regulator in both basal and stress-induced autophagy. In the presence of TMEM189, the ULK1 turnover rate is profoundly increased, which in turn compromises its kinase activity towards the ATG13 and ATG101. Conversely, *TMEM189* knockdown increased the levels of ULK1, which led to increased autophagosome formation during the basal state and following starvation. Furthermore, we found that TMEM189 disrupts ULK1-TRAF6 association, subsequently impairs ULK1 polyubiquitylation by K63-linked chains, self-association and function. Our findings suggest that TMEM189 may play a role in autophagy via two possible mechanisms: (1) TMEM189 may inhibit ULK1 activity and block autophagic progression under physiological conditions, which maintains the homeostasis of basal autophagy; (2) during the autophagic process, a novel regulatory pathway is used, by which cells can avoid excessive self-digestion that culminates in cell death. These findings underscore the importance of TMEM189 in fine-tuning the autophagy-regulating network via ULK1 under physiological conditions.

A number of studies have demonstrated that ULK1 activity is tightly regulated by several post-translational modifications, including ubiquitination. Indeed, E3 ligases have been shown to promote the formation of polyubiquitin chains on substrates through any of the seven lysines (K6, K11, K27, K29, K33, K48, and K63). In addition, Cullin3-KHLH20-mediated ubiquitylation of ULK1 is typically K48-linked, which can degrade ULK1 and downstream substrates (e.g., ATG13, BECN1, and PIK3C3) during prolonged autophagy [10]. Following starvation, NEDD4L ubiquitylates ULK1 using K27- and K29-linked and proteasome degradation, which inhibits the autophagy pathway and subsequently avoids cellular apoptosis induced by excessive autophagy [20]. Thus, such effects may reflect the need for cells to possess alternative backup systems for the termination of autophagy, highlighting the critical requirement for the tight regulation of this event in general cellular homeostasis. In contrast, the molecular chaperone complex, HSP90-Cdc37, and P32 also activates the ULK1 complex and promotes mitochondrial autophagy. Recent studies indicate that ER-localized transmembrane proteins Atlastin 2/3 positively regulate ER targeting of the ULK1 complex to initiate autophagy [21]. Our studies indicate that TMEM189 could impair interaction of ULK1 and TRAF6, abrogate K63-linked polyubiquitylation of ULK1 and self-association, enhance its instability and kinase activities, which resulted in a prominent decline of the levels of phosphorylation of ATG13 at S318. In addition, we also find that TMEM189 negatively regulates the levels of ATG13 and ATG101. Subsequently leads to a prominent decline of the autophagosome biogenesis. We speculate that ER-localized TMEM189 may bind with ULK1, block the recruitment of ATG13 and ATG101, increase the instability of the ULK1 complex in the endoplasmic reticulum, and thus weaken the initiation of autophagy. We will continue our relevant researches on this area.

The relationship between autophagy and cancer is highly complex, as autophagy has been found to exhibit both tumor-suppressive and cancer-promoting functions [22]. In general, autophagy is a mechanism of cellular survival and is thought to function as a tumor suppressor by preventing the accumulation of damaged and/or toxic components that, if left to persist, could lead to genomic instability and cancer. Accumulating evidence indicates that elevated ULK1 expression has been observed in several human cancers, including esophageal squamous cell carcinoma, nasopharyngeal carcinoma, colorectal cancer [23], gastric cancer, hepatocellular carcinoma [24], and clear cell renal carcinoma [25]. It is an independent predictor of poor survival for patients with these cancers. In contrast, low ULK1 expression is associated with operable breast cancer progression and is an adverse prognostic marker of survival for patients [26]. Resent document reported that ULK1 inhibits the metastasis of breast cancer cells by phosphorylating Exo70 protein [27]. The tumor-suppressor roles of Death associated protein kinase 3 (DAPK3) in gastric cancer are associated with increased ULK1 activity and autophagy [28]. These findings indicate that ULK1 may be a tumor suppressor or promoter, depending on the different states of tumorigenesis or tumor development. Cui et al. reported that TMEM189 was strongly correlated with resistance to ferroptosis inducers [15], and suggests TMEM189 as a promising druggable target for anticancer therapy. Using a mouse xenograft model, we showed that *TMEM189* knockdown-triggered autophagy prevents the growth of BGC823 gastric cancer cells, implying a negative correlation between TMEM189 and gastric cancer. In future studies, we will cooperate with clinical laboratories to further clarify the correlation between the expression of TMEM189, ULK1, and autophagy in different tumor patients. Similarly, the physical and functional connection between TMEM189 and ULK1 in response to different conditions will also be elucidated in further experiments.

## Materials and methods

### Antibodies and reagents

The antibodies and major reagents used in this study are listed in Supplementary Table 1.

### Plasmid construction

TMEM189 cDNA was amplified from the cDNA library of HEK293T cells by PCR with the forward primer P1 (5′-CCGGAATTCATGGCGGGCGCCGAGGAC-3′) and the reverse primer P2 (5′-CGCGGATCCCGTTTGATCTTCTGGGCCCATTTC-3′) and cloned into pcDNA.3.1/myc-His(-)B (Invitrogen, V85520) to construct the pcDB-TMEM189 plasmid (TMEM189). The following plasmids expressing tagged TMEM189 were successfully constructed: TMEM189-MYC, FLAG-TMEM189, GFP-TMEM189, GST-TMEM189^1-45^, GST-TMEM189^150-230^ or GST-TMEM189^183-270^. The following plasmids expressing tagged ULK1 (GFP-ULK1, GFP-ULK1-KD^1-278^, GFP-ULK1-S/T^279-827^, GFP-ULK1-CTD^828-1050^), GFP-TRAF6, FLAG-TRAF6, GFP-ZFYVE1/DFCP1, GFP-ATG16L1, FLAG-Ubiquitin and FLAG-UbK63 plasmids were also successfully constructed. The siRNAs against the indicated genes in this study are listed in Supplementary Table 2. All plasmids were confirmed by DNA sequencing.

### Cell culture, transfection, and treatment

HeLa cells, BGC823 cells, and HEK293T cell lines from American Type Culture Collection without mycoplasma contamination were cultured in DMEM (Invitrogen, 12800-017) supplemented with 10% fetal bovine serum and maintained at 37 °C in a humidified chamber containing 5% CO2. The stable GFP-LC3B HeLa cell line was a gift from Li Yu (Tsinghua University, Beijing, China), *ULK1* knockout HeLa cells were from Quan Chen (Nankai University, Tianjin, China), *TRAF6* knockout HEK293T cells were obtained from Zhengfan Jiang (Peking University, Beijing, China). These cells were transfected with the plasmids using MegaTran 1.0 Transfection Reagent (ORIGEN, TT200004), according to the manufacturer’s instructions, while siRNA transfection was performed using Lipofectamine 2000 reagent (Invitrogen, 11668-019). Cellular autophagy was induced by nutrient deprivation through an incubation in EBSS, which contains neither amino acids nor FBS or the MTOR inhibitor, rapamycin (RAPA, 5 μM). Blocking the fusion between autophagosomes and lysosomes was performed by treating cells with 10 μM of BafA1 or 50 μM of chloroquine (CQ). Puromycin was used to select HEK293T, HeLa and BGC823 cell lines that were stably infected with the following lentiviral vectors: pLXV-shRNA control (*shcontrol*) or pLVX-shRNA TMEM189 (*shTMEM189*).

### Immunoprecipitation (IP) and Western blot analysis

For the IP analysis, treated cells were collected and disrupted in RIPA Lysis Buffer containing protease inhibitors (Roche Diagnostics, 04693116001). The total cell extracts (1 mg per sample) were mixed with precleared protein G sepharoseTM Fast Flow (GE Healthcare, 17-0618-01) and the appropriate antibodies, followed by an incubation for 2 h at 4 °C. The beads were collected by centrifugation, washed five times, resuspended in 2 × SDS loading buffer, and analyzed by Western blotting. The protein bands were visualized using DyLight 800/DyLight 680-conjugated secondary antibodies, and the infrared fluorescence image was obtained using an Odyssey infrared imaging system (LI-CORBiosciences, Lincln, NE, USA).

### GST affinity isolation assay

Recombinant GST, GST-TMEM189^1-45^, GST-TMEM189^150-230^, or GST-TMEM189^183-270^ fusion proteins were expressed in Escherichia coli strain BL21 (DE3) and purified. Equal amounts of these proteins were mixed with the whole cell lysates extracted from the indicated plasmids transfected cells and glutathione-SepharoseTM4B (GE Healthcare, 17-0756-01) for 2 h at 4 °C. After five washes, the beads were resuspended in 2 × SDS loading buffer and analyzed by Western blotting.

### Immunofluorescence and confocal microscopy assays

Cells were cultured in confocal dishes and transfected with the indicated plasmids, then washed with PBS and fixed in 4% PFA (dissolved in PBS) for 30 min at 37 °C. The fixed cells were permeabilized with 0.2% Triton X-100 for 15 min at 37 °C and blocked with BSA for 1 h. These cells were then incubated with primary antibodies overnight at 4 °C. After washing three times, cells were stained with FITC-conjugated secondary antibodies and imaged by a Leica TCSSP8 Confocal Microscope.

### Real-time quantitative PCR (RT-qPCR)

Total RNA samples were extracted with the TRIzol reagent (Invitrogen, 15596-026), then were reverse transcribed using the ThermoScript RT-PCR system (Invitrogen Life Technologies) according to the manufacturer’s protocol. All gene transcripts were quantified by real-time PCR with SYBR green qPCR Mix kit (Genstar). The primers used for RT-qPCR were listed in Supplementary Table 2.

### Cell viability and colony formation assays

The stable *shcontrol*- or *shTMEM189*-expressing BGC823 cells were treated with serum deprivation for 18 h. Cells were then cultured in complete culture medium for indicated time. Cell viability assays were performed using the CellTiter 96 AQueous One Solution Cell Proliferation Assay (Promega, G1111) according to the manufacturer’s instructions. Absorbance at 490 nm was measured on an EL-311SX ELISA Reader (Bio-Tec Instruments, USA). Cell viability was calculated as follows: cell viability = absorbance of test group/absorbance of control cell group × 100%. Each experiment was performed in biological triplicate and independently repeated three times.

For the colony formation assay, The stable *shcontrol*- or *shTMEM189*-expressing BGC823 cells were plated in triplicate at 100 or 200 cells/well, and cultured for 10~14 days in 24-cell plates. Then cells were fixed with methanol for 15 min and stained with crystal violet for 30 min. Colonies were counted and photographed.

### Tumorigenicity in nude mice

A nude mouse xenograft model was established using six to eight-week-old male BALB/c nude mice (Experimental Animal Center, Peking University Health Sciences Center, Beijing, China). The mice were housed and maintained in a pathogen-free facility, and all experimental procedures were approved by the Institutional Authority for Laboratory Animal Care of Peking University (LA2018033). *shcontrol/*BGC823 or *shTMEM189/*BGC823 cells were subcutaneously injected into the right axilla of the BALB/c nude mice in a total volume of 100 μL (4 × 10^6^ cells/mouse, *n* = 6). Mice were sacrificed at 22 days after the cell inoculation, at which time the tumors were excised and photographed.

### Statistical analysis

Data are shown as the mean ± SD. Statistical differences between groups were analyzed using Student’s *t* test for continuous variables, thus *P* < 0.05 was regarded as statistical significance. All statistical analyses results were performed with GraphPad Prism^®^ software.

## Supplementary information

Supplementary Table1

Supplementary Table 2

Supplementary figures and figure legends

Oiginal Western Blot Figures

Checklist

## Data Availability

The data can be made available upon reasonable request to the corresponding author at the following address: Yingyu Chen, Department of Immunology, Peking University School of Basic Medical Sciences, 38 Xueyuan Road, Beijing, 100191, China. Email: yingyu_chen@bjmu.edu.cn.
