# Supplementary material for: TMEM189 negatively regulates the stability of ULK1 protein and cell autophagy
Source: Cell Death Dis. 2022 Apr 7;13(4):316. doi: 10.1038/s41419-022-04722-y (PMC8991247; doi:10.1038/s41419-022-04722-y)
Supplement: Supplementary file 1 — Supplementary Table1 [file 41419_2022_4722_MOESM1_ESM.docx]

| Table.1 List of antibodies and reagents used in this manuscript | | |
| --- | --- | --- |
| Name | Art. No of the Products | Company |
| Anti-LC3B | L7543 | Sigma Aldrich |
| Anti-TMEM189 | T8953 |  |
| anti-ULK1 | 8054S | Cell Signaling technology |
| Anti-ATG13 | 13468 |  |
| Anti-Phospho-ATG13 (Ser318) | 43533S |  |
| anti-p62/SQSTM1 | 5114 |  |
| anti-ubiquitin | 3936 |  |
| Anti-ATG101 | 13429S |  |
| Anti-ATG14 | 5504S |  |
| Anti-Phospho-ATG14 (Ser29) | 13155S |  |
| Anti-BECN1 | PD017 |  |
| Anti-Phospho-BECN (Ser15) | 13825S |  |
| Anti-FIP200 | AB176816 | Abcam |
| Anti-ACTB/β-actin | KM9001 | Tianjin Sungene Biotech |
| Anti-HA | KM8004 |  |
| Anti-GFP | KM8009L |  |
| Anti-GST | KM8005 |  |
| Anti- MYC | KM8003 |  |
| Anti- FLAG | KM8002 |  |
| Anti-GAPDH | KM9002 |  |
| DyLight 800/DyLight 680-conjugated secondary antibodies against rabbit | 611-145-002/611-144-002 | Rockland |
| DyLight 800/DyLight 680-conjugated secondary antibodies against mouse | 610-145-002/610-144-002 |  |
| FITC/RBITC-conjugated secondary antibodies against mouse | bs-0296G-FITC/bs-0296G-RBITC | Bioss Inc. |
| FITC/RBITC -conjugated secondary antibodies against rabbit | bs-0295G-FITC/bs-0295G-RBITC/ |  |
| EBSS（Earles’s Blanced salts solution） | 24010043 | Invitrogen |
| Bafilomycin A1 (Baf.A1) | B1793 | Sigma Aldrich |
| Rapamycin (RAPA) | R117 |  |
| Chloroquine (CQ) | C6628 |  |
| MG132 | C2211 |  |
| Hoechst 33342 | 14533 |  |
| Cycloheximide | 239763 | Calbiochem |
