# Supplementary material for: TMEM189 negatively regulates the stability of ULK1 protein and cell autophagy
Source: Cell Death Dis. 2022 Apr 7;13(4):316. doi: 10.1038/s41419-022-04722-y (PMC8991247; doi:10.1038/s41419-022-04722-y)
Supplement: Supplementary file 2 — Supplementary Table 2 [file 41419_2022_4722_MOESM2_ESM.docx]

## **Supplementary Table 2. The siRNA and Primer sequences used in this study**

| Name | | Sequence |
| --- | --- | --- |
| Control *siRNA* | | 5’- UUCUCCGAACGUGUCACGUTT-3’ |
| *TMEM189 siRNA-1* | | 5’- GCAUCACCACAGGCUGGCUCACTT -3’ |
| *TMEM189 siRNA-2* | | 5’- UGACAUGAAAUGGGCCCAGAATT-3’ |
| RT-PCR | |  |
| *TMEM189* | Forward Primer | 5’- GAGGACGAGGCGTCTTGTTG -3’ |
|  | Reverse Primer | 5’- CAGCACCCCAGTGTACCAG -3’ |
| *GAPDH* | Forward Primer | 5’- GAAGGTGAAGGTCGGAGTC -3’ |
|  | Reverse Primer | 5’- GAAGATGGTGATGGGATTTC -3’ |
| qRT-PCR | |  |
| *TMEM189* | Forward Primer | 5’- CCACGTAAACACCATCGCAT -3’ |
|  | Reverse Primer | 5’- TCTGGGCCCATTTCATGTCA -3’ |
| *GAPDH* | Forward Primer | 5’- TCAAGAAGGTGGTGAAGCAGG -3’ |
|  | Reverse Primer | 5’- TCAAAGGTGGAGGAGTGGGT -3’ |
