# Supplementary material for: TMEM189 negatively regulates the stability of ULK1 protein and cell autophagy
Source: Cell Death Dis. 2022 Apr 7;13(4):316. doi: 10.1038/s41419-022-04722-y (PMC8991247; doi:10.1038/s41419-022-04722-y)
Supplement: Supplementary file 3 — Supplementary figures and figure legends [file 41419_2022_4722_MOESM3_ESM.docx]

**Supplementary data**

**TMEM189 negatively regulates the stability of ULK1** **complex and cell autophagy**

Jiahong Yu^1,6^, Liujing Qu^1,2,6^, Yan Xia^1,6^, Xuan Zhang^1^, Jinqiu Feng^1^, Mengyuan Duan^1^, Pengli guo^1^, Yaxin Lou^3^, Ping Lv^1^, Wenping Lv^4,^*, Yingyu Chen^1,5^*

^1^ Department of Immunology, Peking University School of Basic Medical Sciences; NHC Key Laboratory of Medical Immunology, Peking University, 38 Xueyuan Road, Beijing, 100191, China.

^2^ Department of Clinical Laboratory, The Affiliated Yantai Yuhuangding Hospital of Qingdao University, 20 Yuhuangding East Road, Yantai, Shandong Province, 264000, China.

^3^ Medical and Healthy Analytical Center, Peking University, 38 Xueyuan Road, Beijing, 100191, China.

^4^ Department of Hepatobiliary Surgery, First Medical Center, Chinese PLA General Hospital, 28 Fuxing Road, Beijing, 100853, China.

^5^ Center for Human Disease Genomics, Peking University, Beijing, 38 Xueyuan Road, Beijing, 100191, China.

^6^ These authors contributed equally to this work.

* Corresponding authors. Email addresses: [lvwenping301@126.com](mailto:lvwenping301@126.com), [yingyu_chen@bjmu.edu.cn](mailto:yingyu_chen@bjmu.edu.cn)

**Running title:** TMEM189 negatively regulates autophagy

**Supplementary Figures and Figure Legends**

**
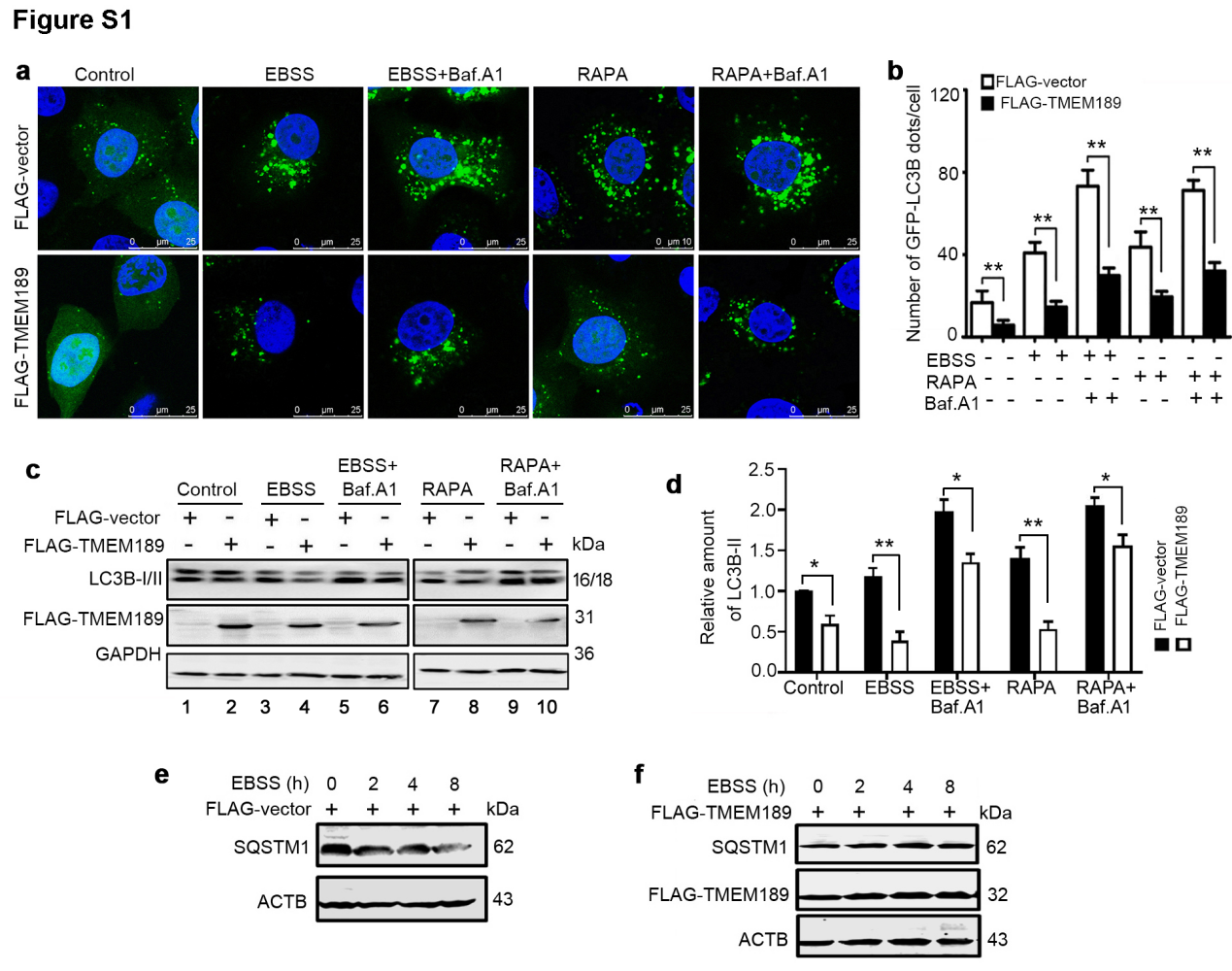
**

**Fig. S1 TMEM189 overexpression decreases autophagosome formation. (a)**The stable GFP-LC3B-expressing HeLa cells were transfected with FLAG-vector or FLAG-TMEM189 for 24 h, then treated with or without BafA1 (10 nM) and/or RAPA (5 μM) for 4 h, or incubated in EBSS for 2 h. The GFP-LC3B dot distribution was observed by confocal microscopy. (**b**) Quantification of GFP-LC3B dots per cell was counted. Data are means ± SD of at least 50 cells. (**c**) Hela cells transfected with the indicated plasmids and treated as in (a), the levels of endogenous LC3B-II were detected by immunoblotting. (**d**) Quantification of amounts of LC3B-II relative to ACTB in cells. Average value in vector-transfected cells (lane 1) was normalized as 1. Data are means ± SD of results from 3 experiments**. (e** and **f)** HeLa cells were transfected with indicated plasmids for 24 h, then incubated in EBSS for indicated time, the levels of SQSTM1/P62 were analyzed by immunoblotting. **P* < 0.05, ** *P* < 0.01.


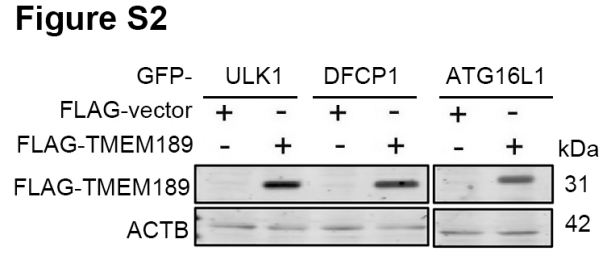


**Fig. S2** FLAG-TMEM189 overexpression in HeLa cells. HeLa cells were transfected with indicated plasmids for 24 h, the levels of FLAG-TMEM189 were detected by western blotting.


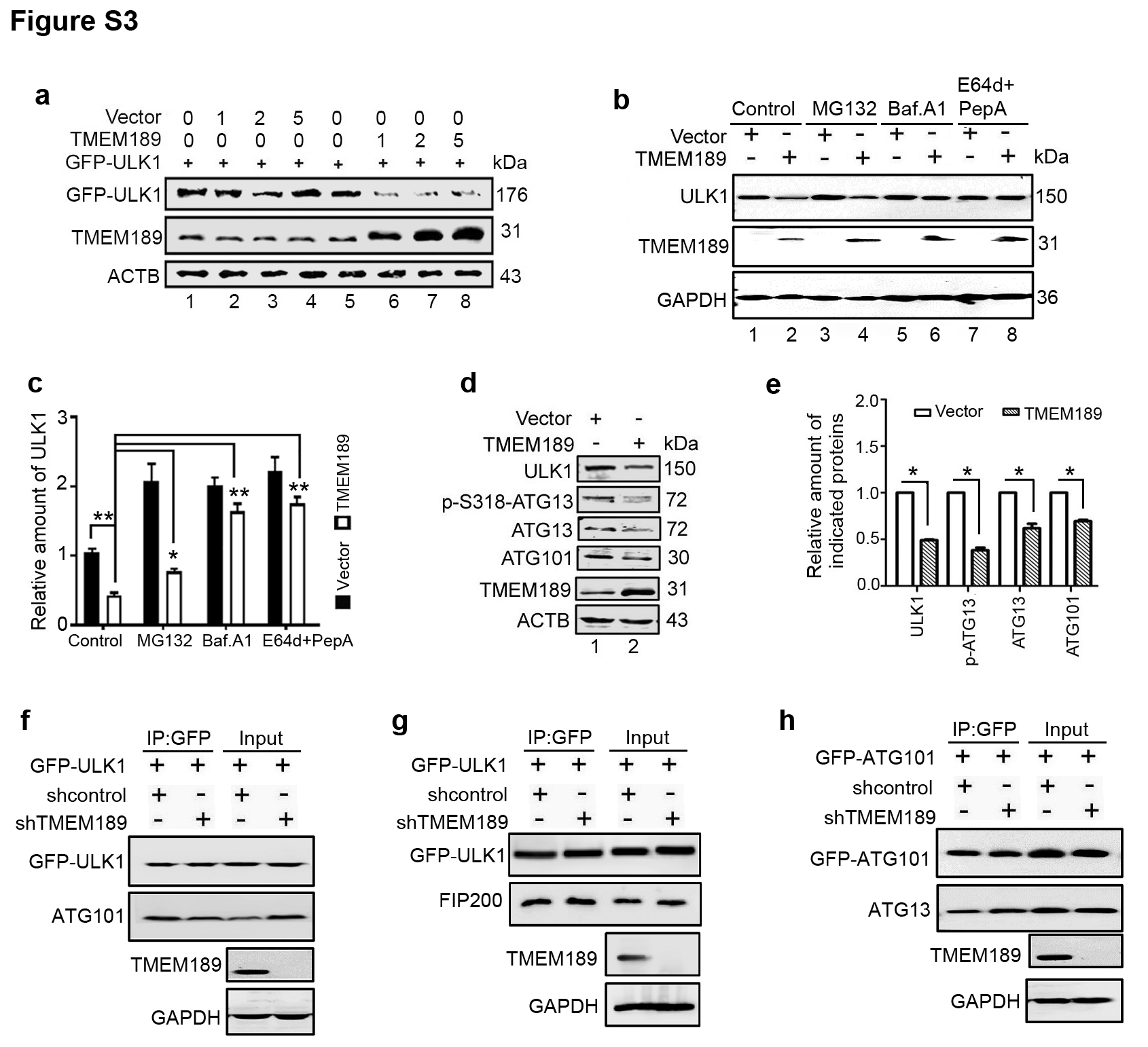


**Fig. S3 TMEM189 negatively regulates stability and kinase activities of ULK1 complex**. (**a**) HEK293T cells were cotransfected with indicated plasmids for 24 h, the levels of GFP-ULK1 were detected by immunoblotting. (**b**) HEK293T cells were transfected with indicated plasmids for 24 h, then treated with MG132 (20μM) for 6 h, Baf.A1 (10 nM) or E64d+PepA for 4 h. The levels of ULK1 and TMEM189 were detected by western blotting. (**c**) Quantification of amounts of ULK1 relative to GAPDH in cells. Average value in vector-transfected cells (lane 1) was normalized as 1, data are means ± SD of results from 3 experiments. (**d**) HEK293T cells were transfected with indicated plasmids for 24 h, the levels of endogenous ULK1, ATG13, p-S318-ATG13 and ATG101 were detected by immunoblotting. (**e**) Quantification of amounts of indicated proteins relative to ACTB in cells. Average value in vector-transfected cells was normalized as 1. Data are means ± SD of results from 3 experiments. (**f-h**) The stable *shcontrol*- or *shTMEM189*-expressing HeLa cells were transfected with ULK1-GFP or GFP-ATG101. Cell lysates were immunoprecipitated using an anti-GFP and analyzed by immunoblotting with anti-ATG101, anti-FIP200 or anti-ATG13 antibodies. Simultaneously, 10% cell lysates were used to immunoblotting. **P* < 0.05, ***P*<0.01.


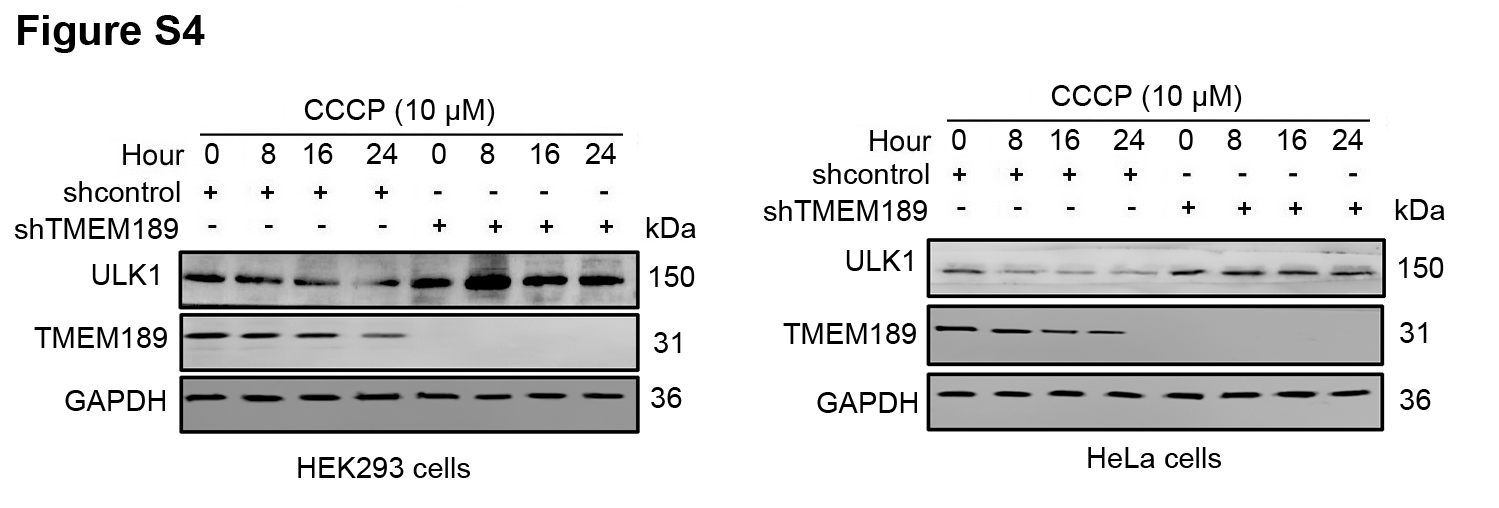


**Fig. S4 Knockdown of *TMEM189* enhances the stability of ULK1 protein.** The stable shcontrol- or shTMEM189-expressing cells were incubated with CCCP for indicated time. The levels of endogenous ULK1, TMEM189 and GAPDH were detected by immunoblotting.


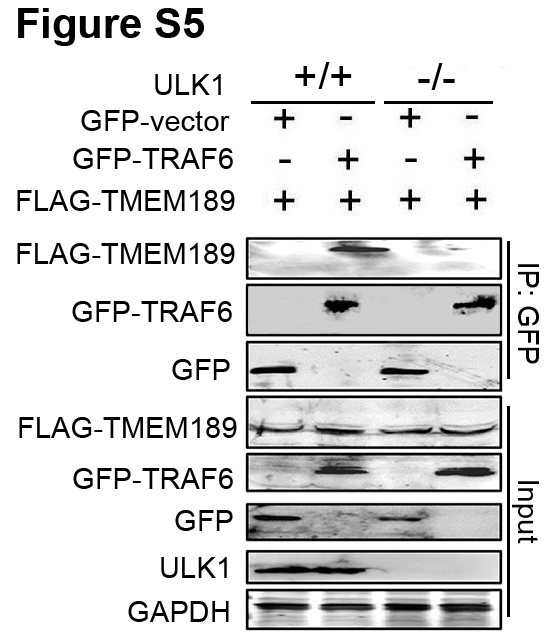


**Fig. S5 The interaction of TMEM189-TRAF6 depends on the presence of ULK1.** *ULK1* wild-type (*ULK1^+/+^*) and *ULK1* knockout (*ULK1^-/-^*) HeLa cells were cotransfected with indicated plasmids for 24 h, then cell lysates were subjected to IP using an anti-GFP. GFP-TRAF6 and FLAG-TMEM189 proteins were detected in the immunoprecipitates by immunoblotting. Simultaneously, 10% cell lysates were used to immunoblotting.


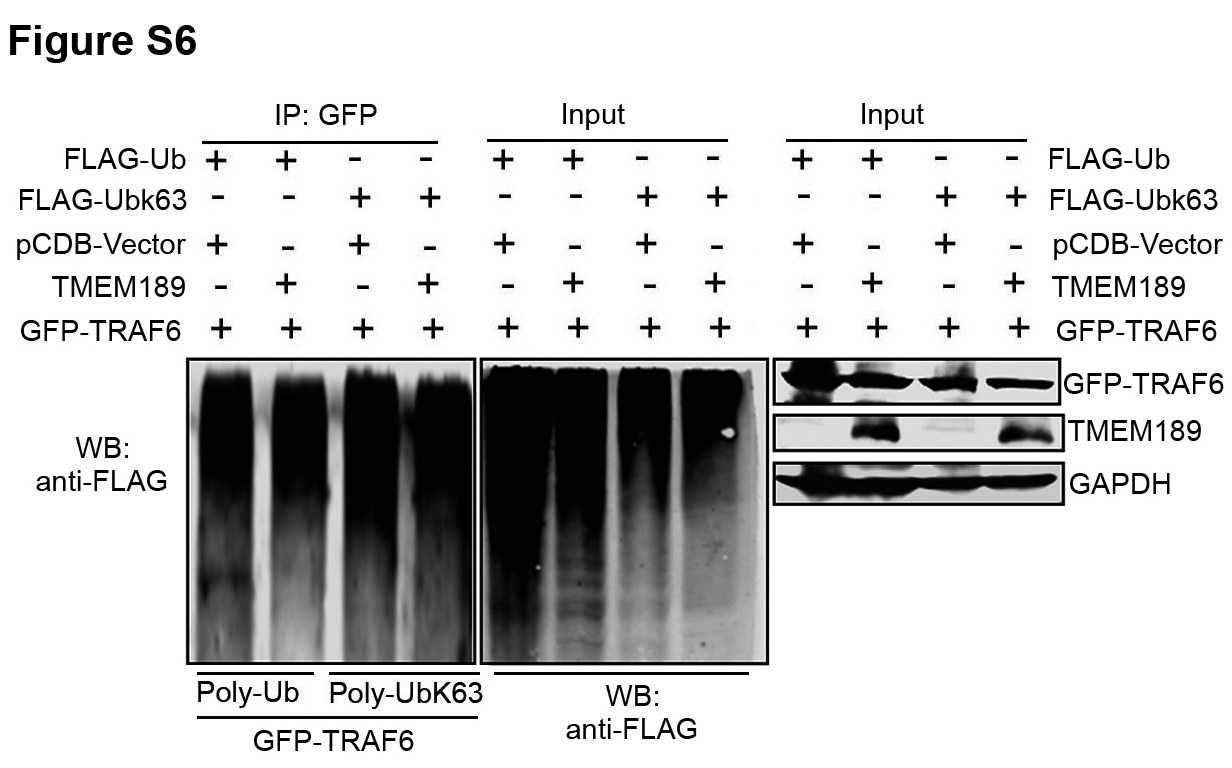


**Fig. S6 TMEM189 failed to affect TRAF6-linked K63 polyubiquitylation.** HEK293T cells were cotransfected with indicated plasmids for 24 h, then treated with MG132 for 6 h. These cell lysates were subjected to IP using an anti-GFP, and the immunoblotting was probed with an anti-FLAG antibody to detect polyubiquitinated GFP-TRAF6 (left panel). Simultaneously, 10% cell lysates were used to immunoblotting.


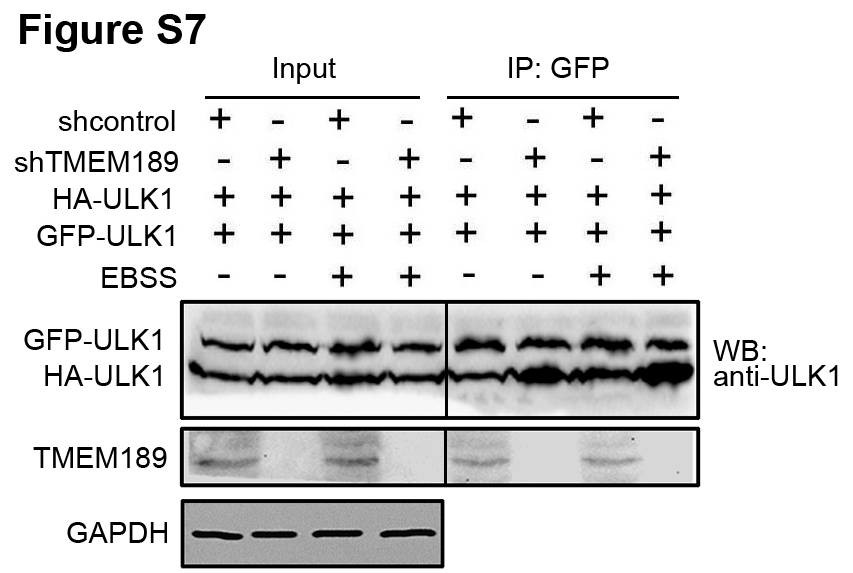


**Fig. S7 Knockdown of TMEM189 increases ULK1 self association.** The stable *shcontrol*- or *shTMEM189*-expressing HeLa cells were transfected with indicated plasmids for 24 h with or without EBSS incubation for 30 min. Then cell lysates were subjected to IP using an anti-GFP. HA-ULK1, GFP-ULK1 and TMEM189 proteins were detected in the immunoprecipitates by immunoblotting. Simultaneously, 10% cell lysates were used to immunoblotting.


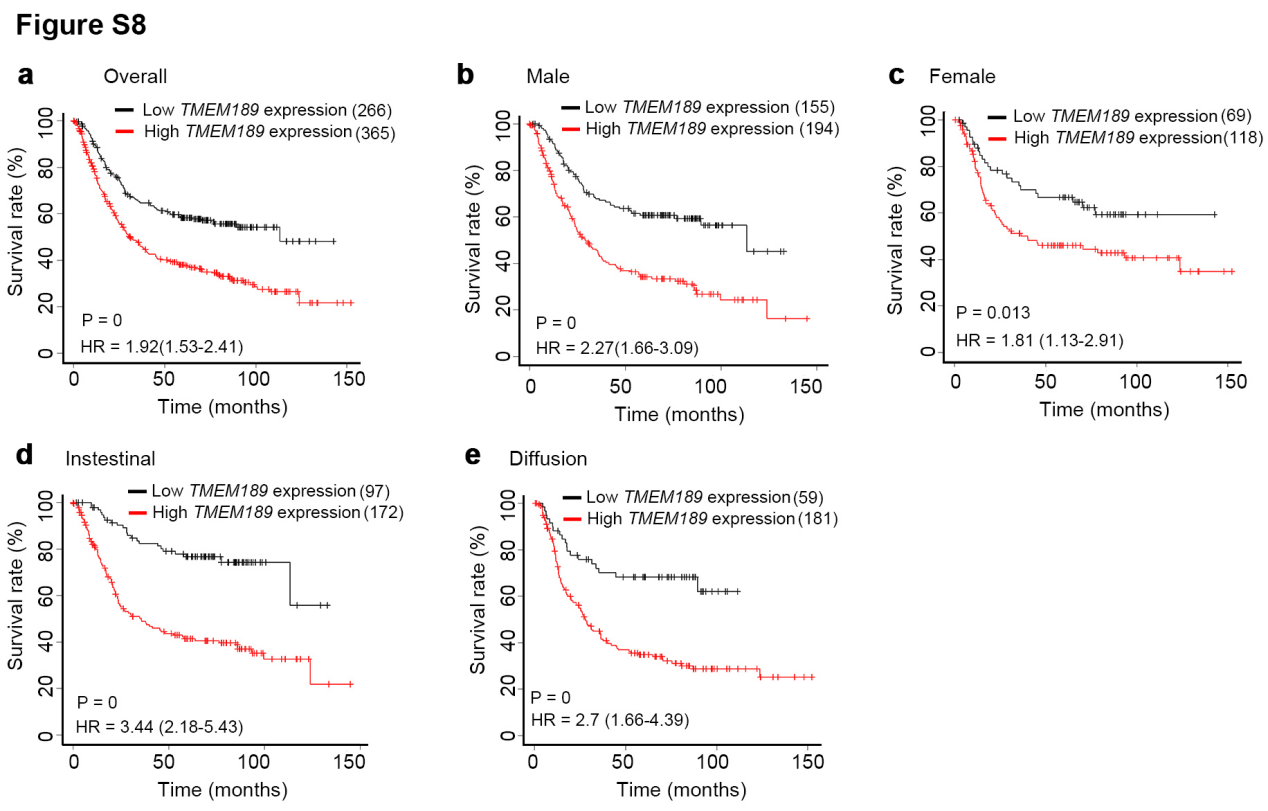


**Fig. S8. The correlation between the levels of *TMEM189* mRNA and the survival time in patients with gastric cancer.**
