# Supplementary material for: TMEM189 negatively regulates the stability of ULK1 protein and cell autophagy
Source: Cell Death Dis. 2022 Apr 7;13(4):316. doi: 10.1038/s41419-022-04722-y (PMC8991247; doi:10.1038/s41419-022-04722-y)
Supplement: Supplementary file 4 — Oiginal Western Blot Figures [file 41419_2022_4722_MOESM4_ESM.pptx]

## Slide 1
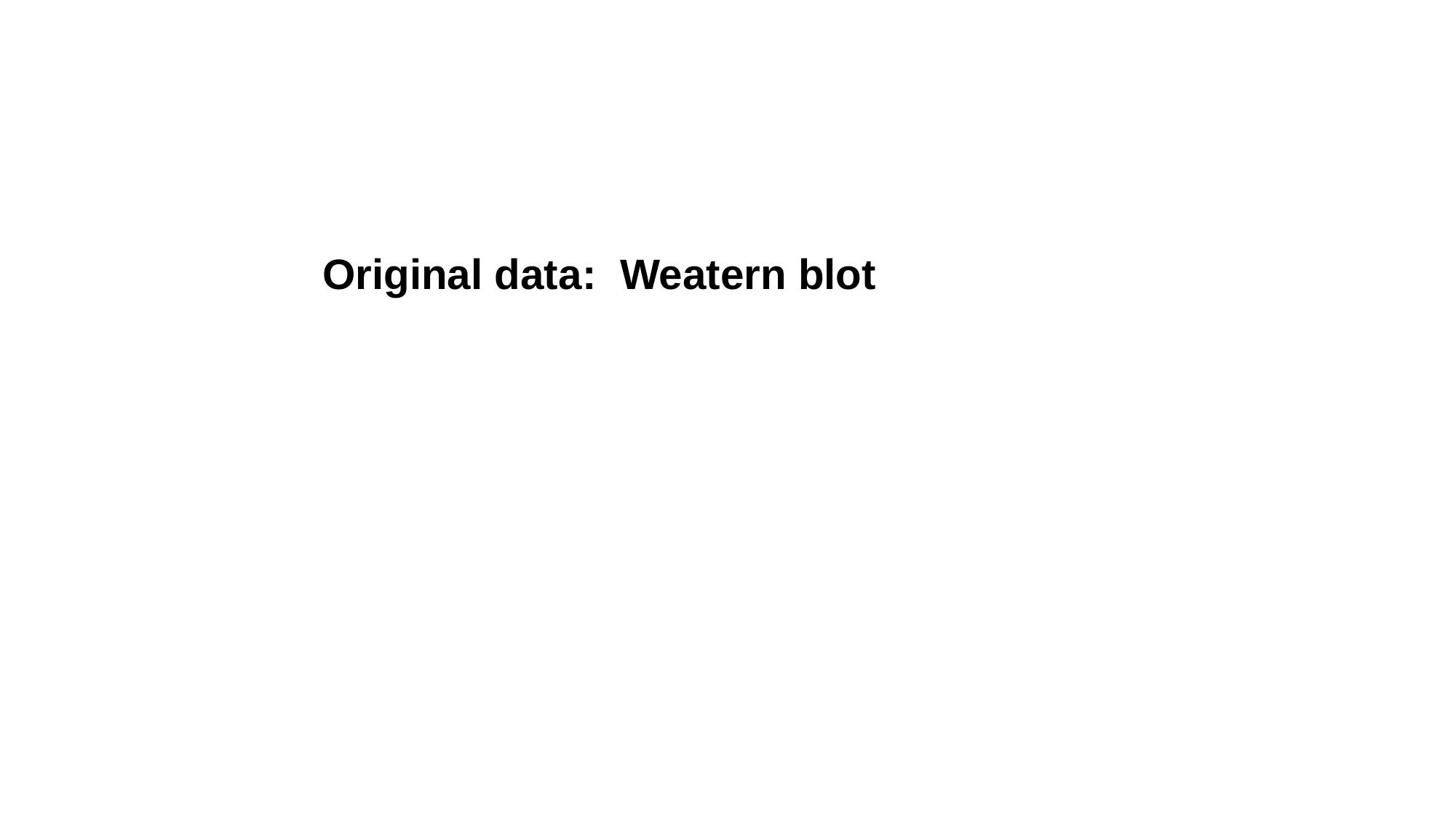

Original data: Weatern blot

## Slide 2
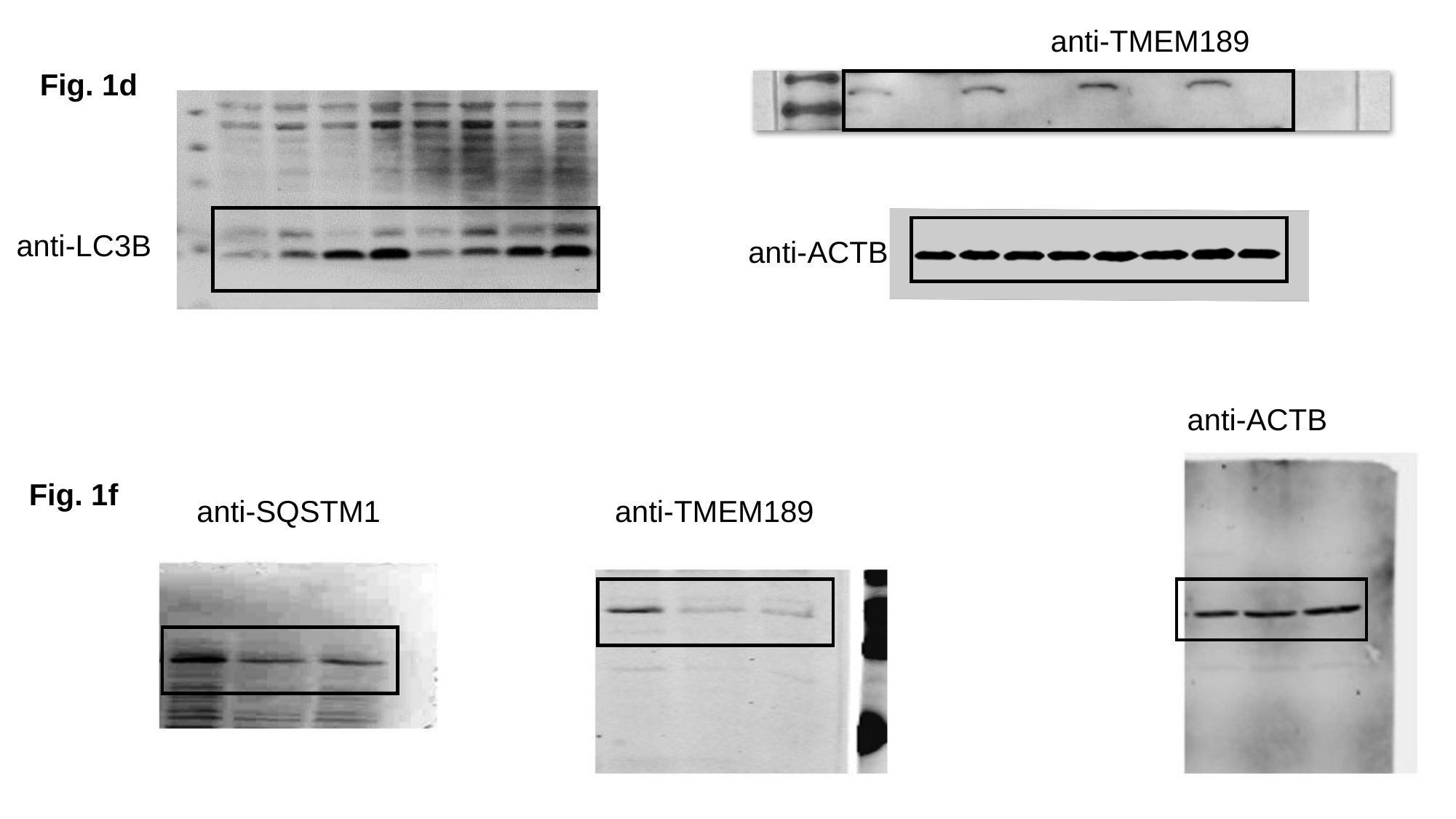

anti-TMEM189
Fig. 1d
anti-LC3B
anti-ACTB
anti-ACTB
Fig. 1f
anti-SQSTM1 anti-TMEM189

## Slide 3
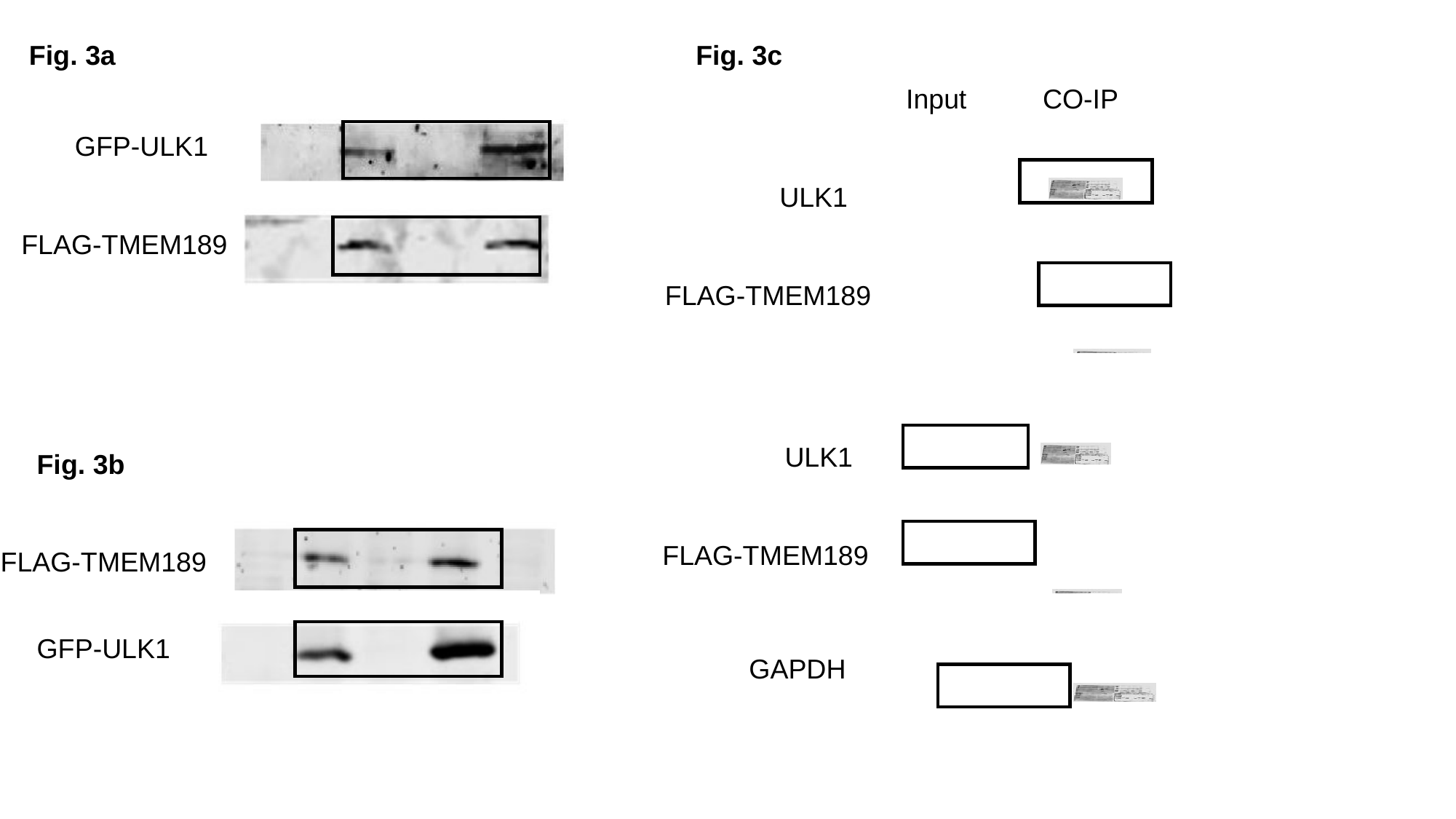

Fig. 3a
 Fig. 3c
Input CO-IP
 GFP-ULK1
FLAG-TMEM189
 ULK1
FLAG-TMEM189
 ULK1
FLAG-TMEM189
Fig. 3b
FLAG-TMEM189
GFP-ULK1
GAPDH

## Slide 4
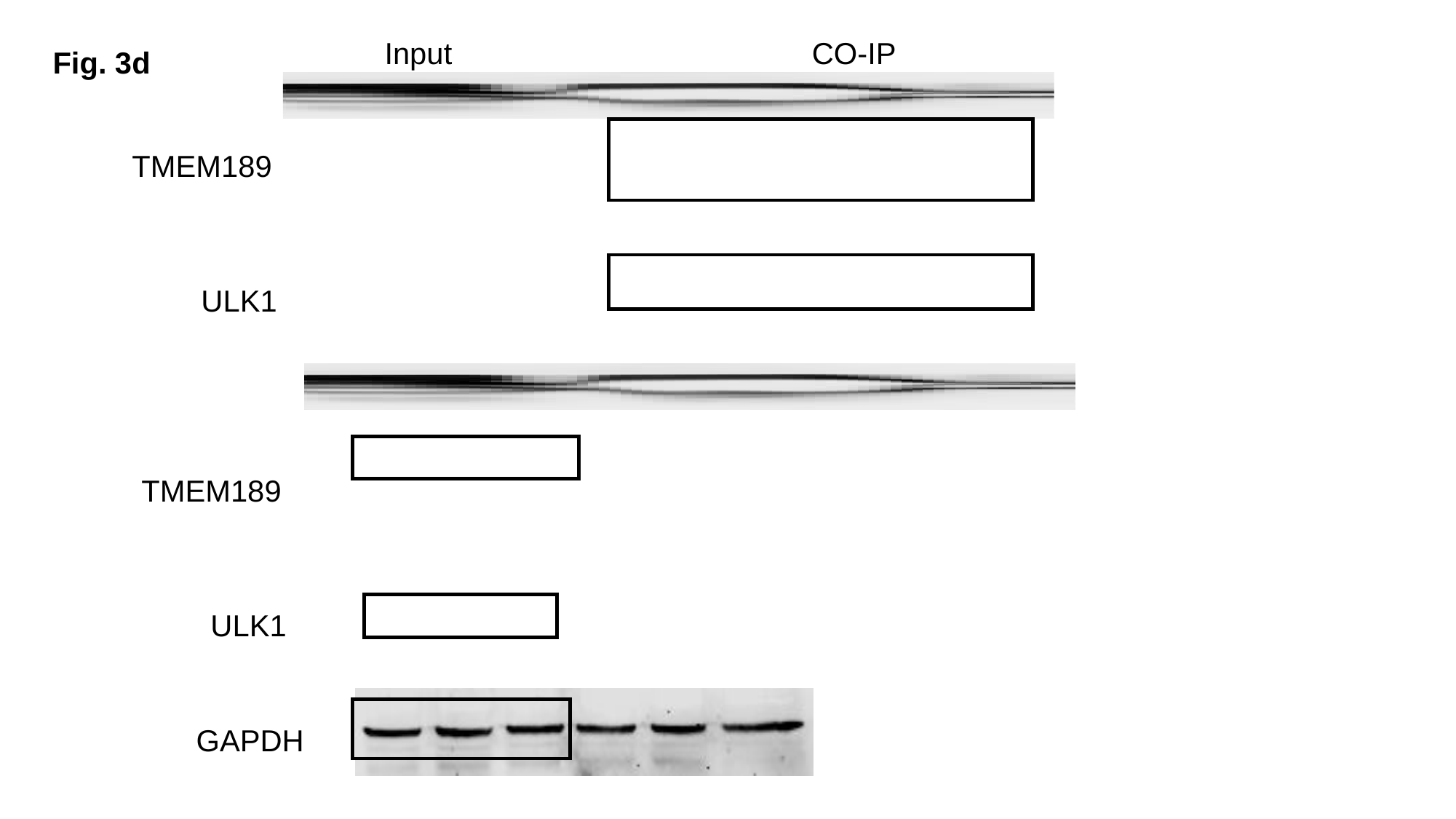

Input CO-IP
 Fig. 3d
TMEM189
ULK1
TMEM189
ULK1
GAPDH

## Slide 5
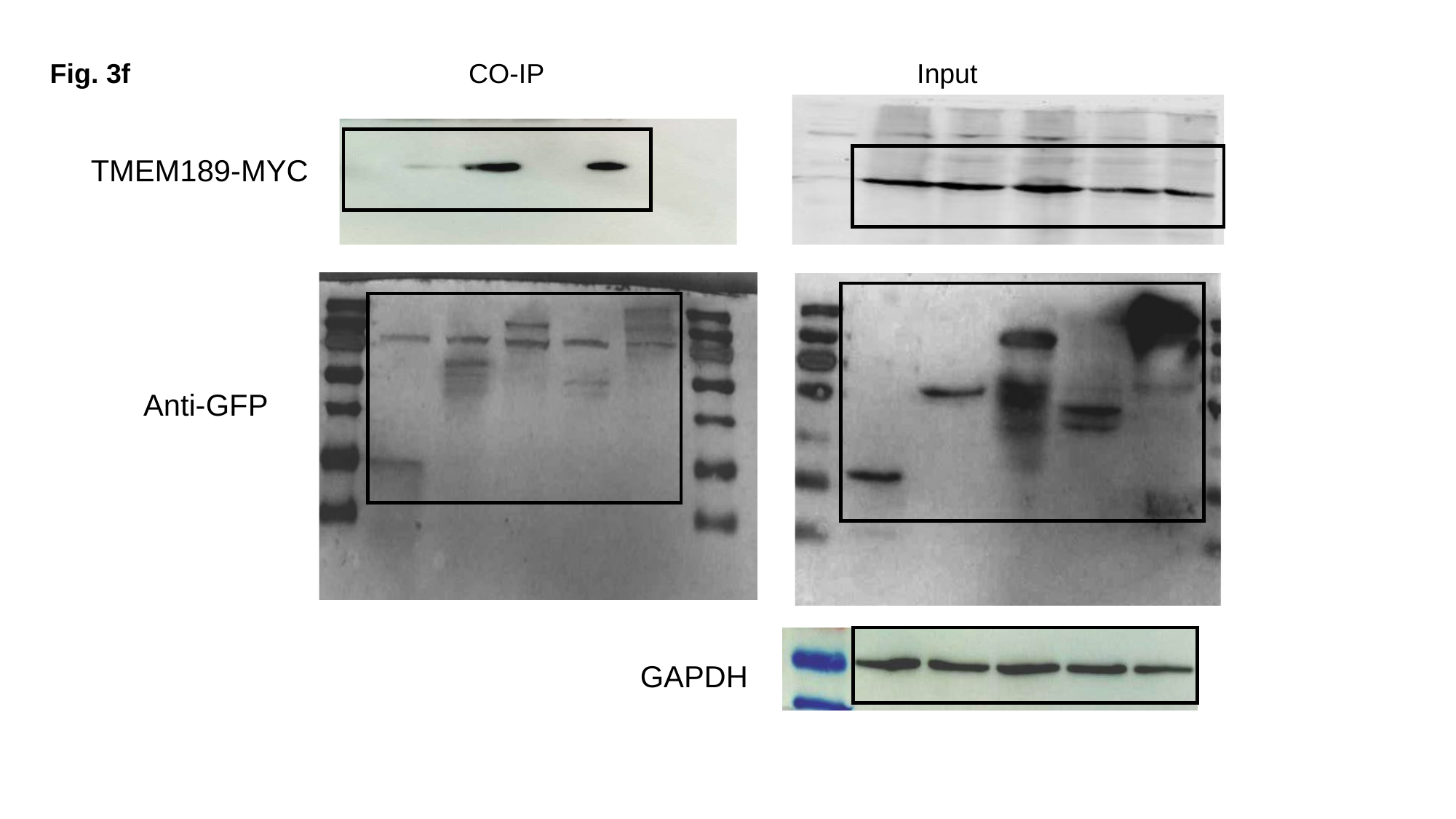

CO-IP Input
 Fig. 3f
TMEM189-MYC
B
| Anti-GFP |
| --- |
| GAPDH |
| --- |

## Slide 6
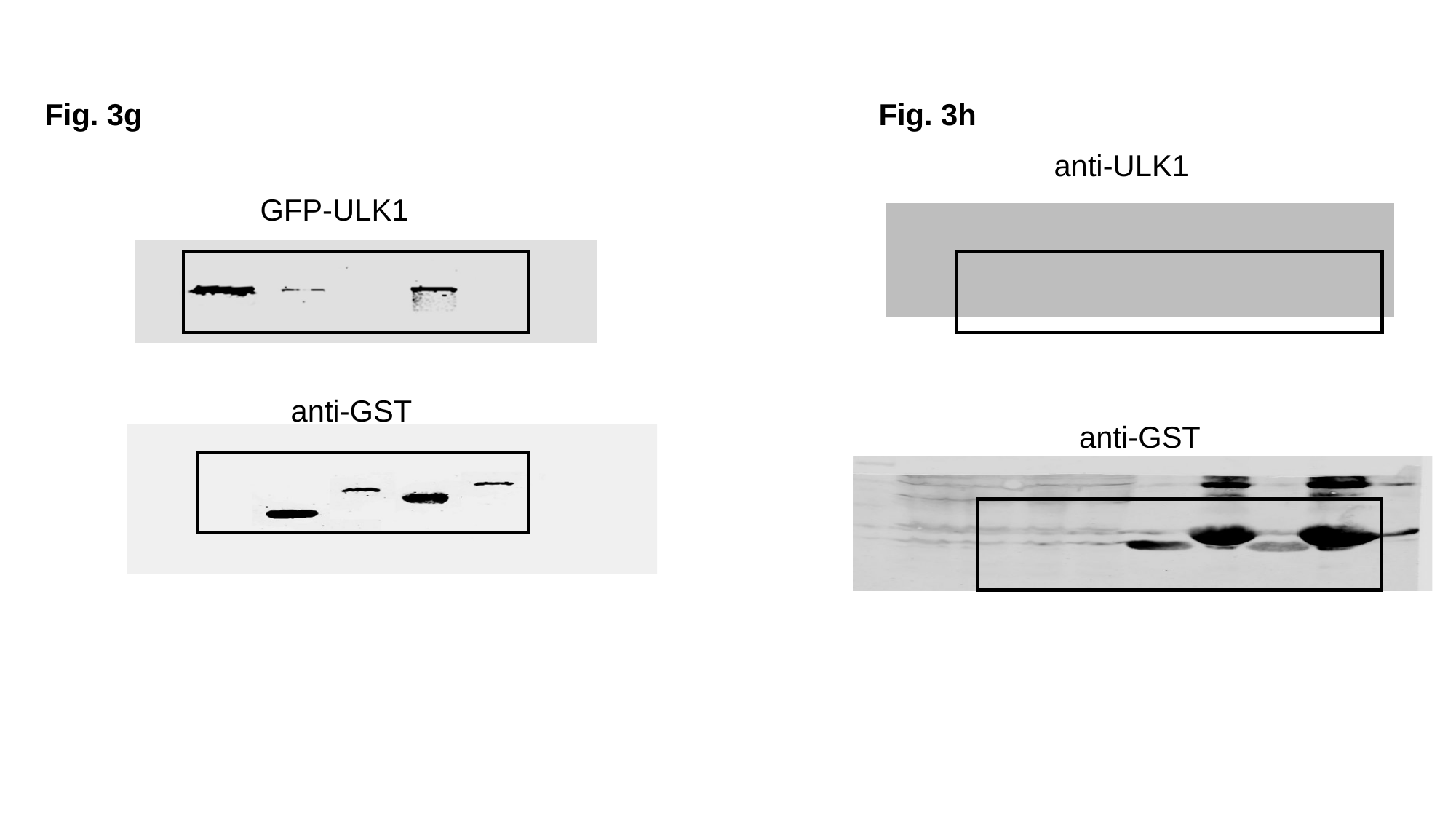

Fig. 3g Fig. 3h
anti-ULK1
GFP-ULK1
anti-GST
anti-GST

## Slide 7
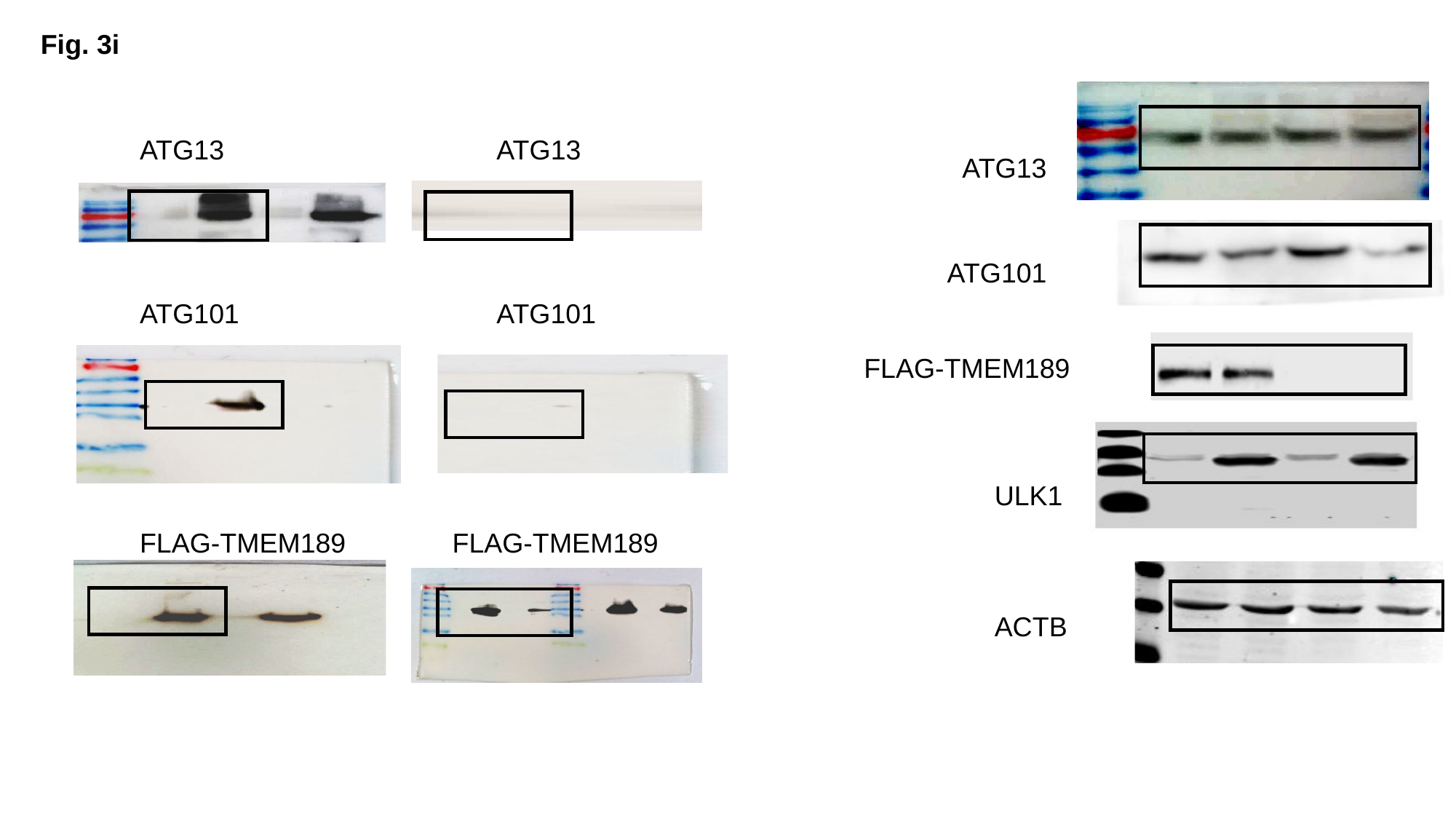

Fig. 3i
ATG13 ATG13
ATG101 ATG101
FLAG-TMEM189 FLAG-TMEM189
ATG13
ATG101
FLAG-TMEM189
ULK1
ACTB

## Slide 8
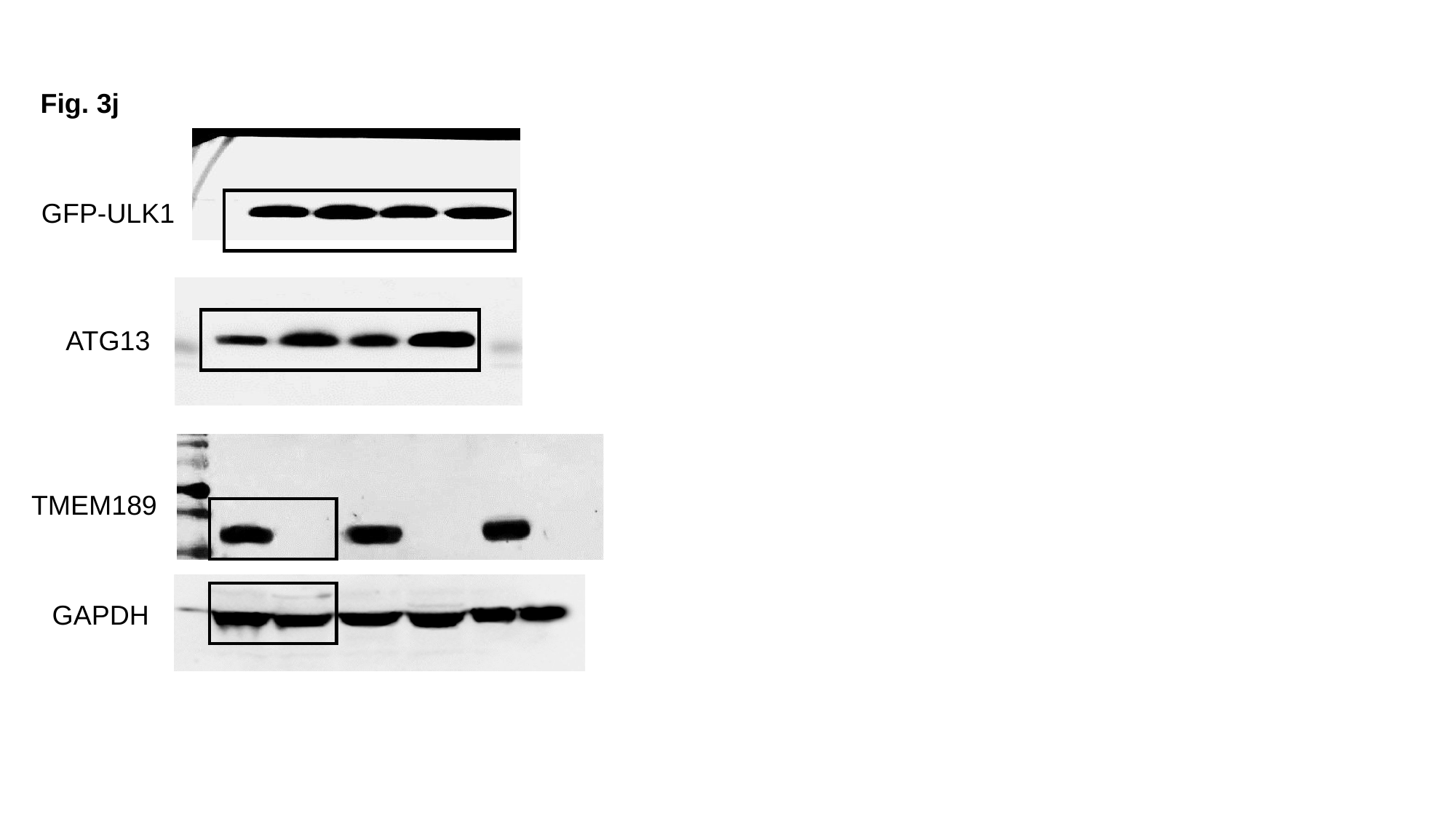

Fig. 3j
GFP-ULK1
ATG13
TMEM189
GAPDH

## Slide 9
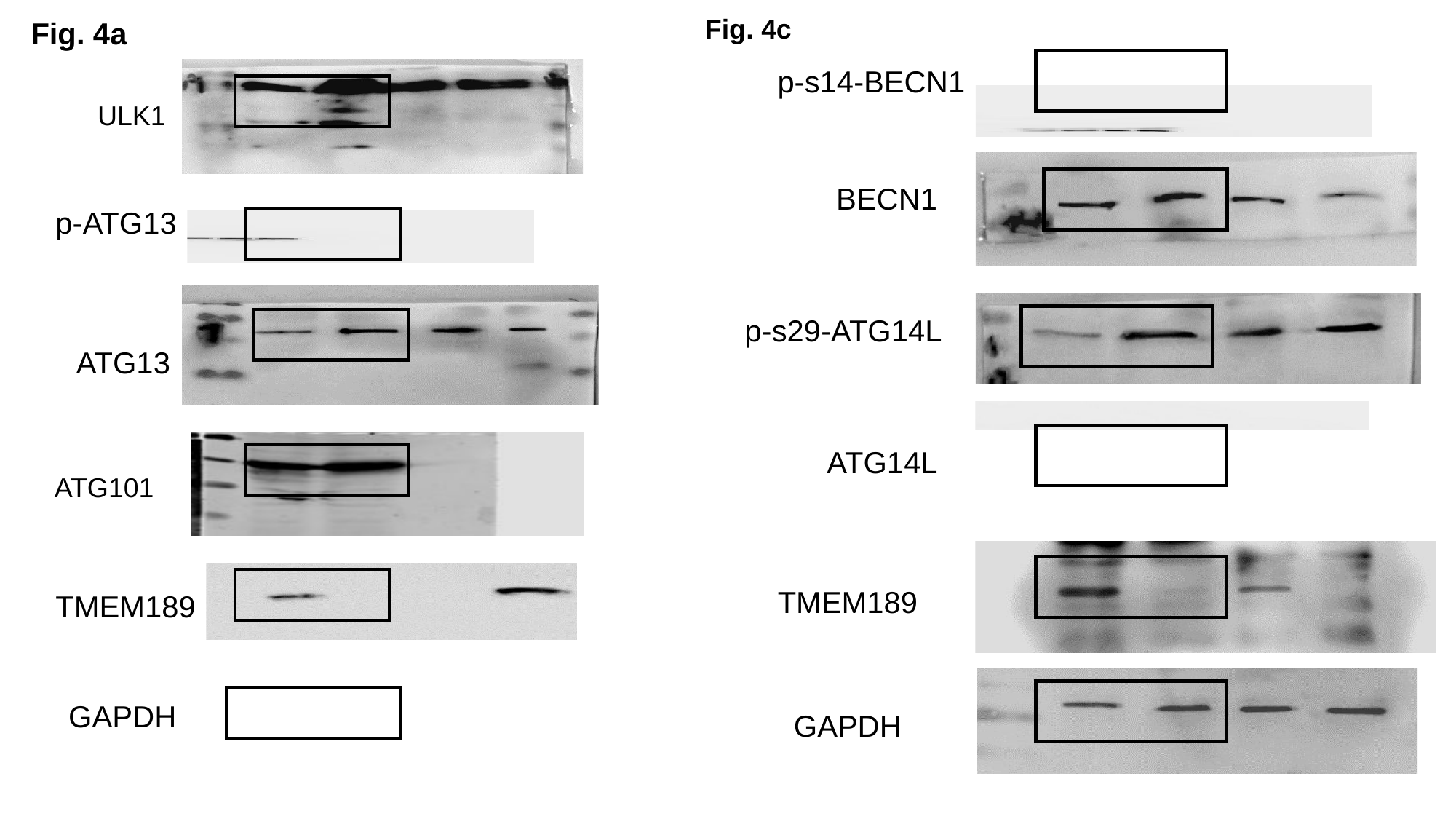

Fig. 4c
Fig. 4a
p-s14-BECN1
ULK1
BECN1
p-ATG13
p-s29-ATG14L
| |
| --- |
| |
ATG13
| |
| --- |
| |
ATG14L
ATG101
TMEM189
TMEM189
GAPDH
GAPDH

## Slide 10
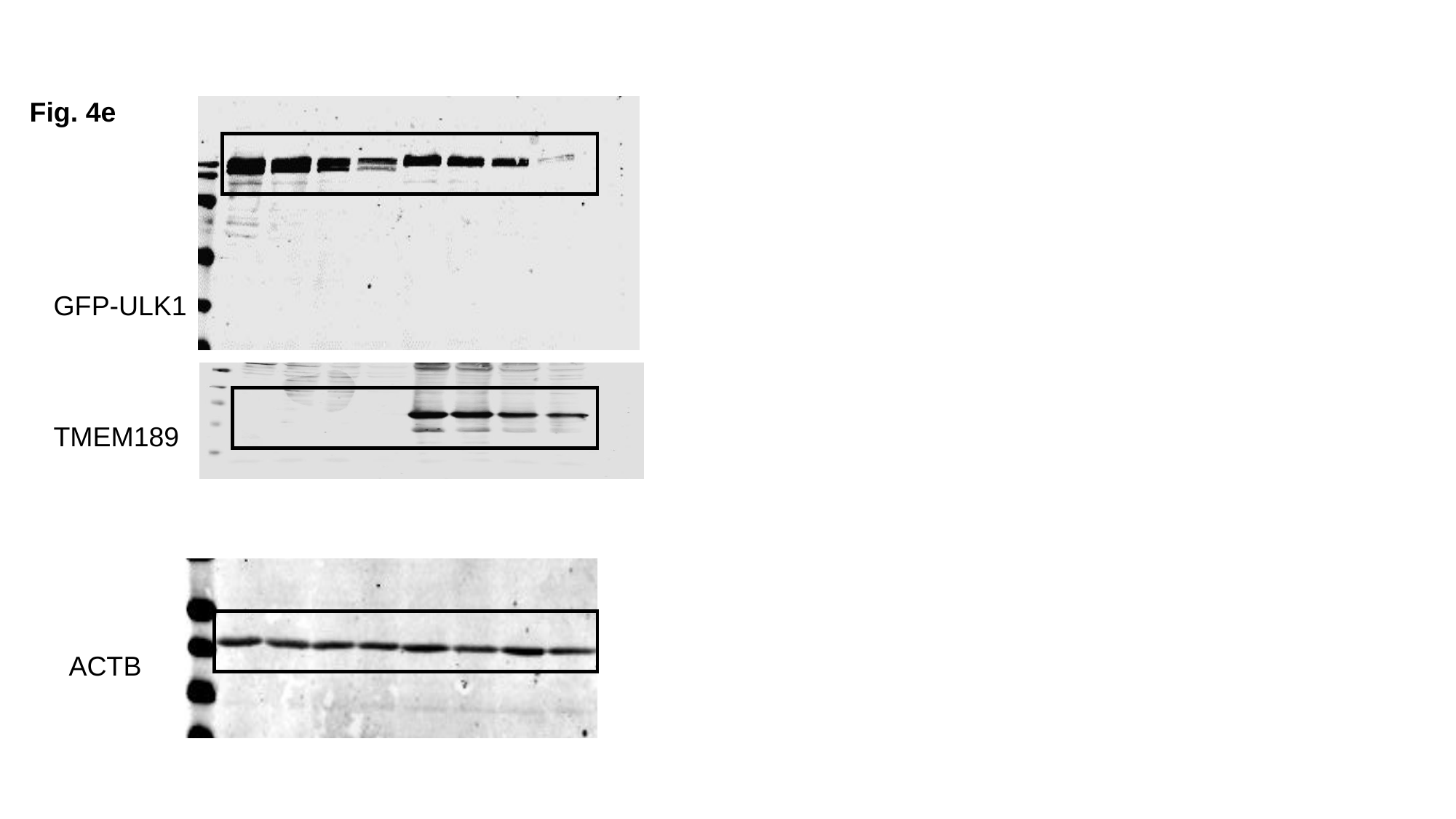

Fig. 4e
GFP-ULK1
TMEM189
 ACTB

## Slide 11
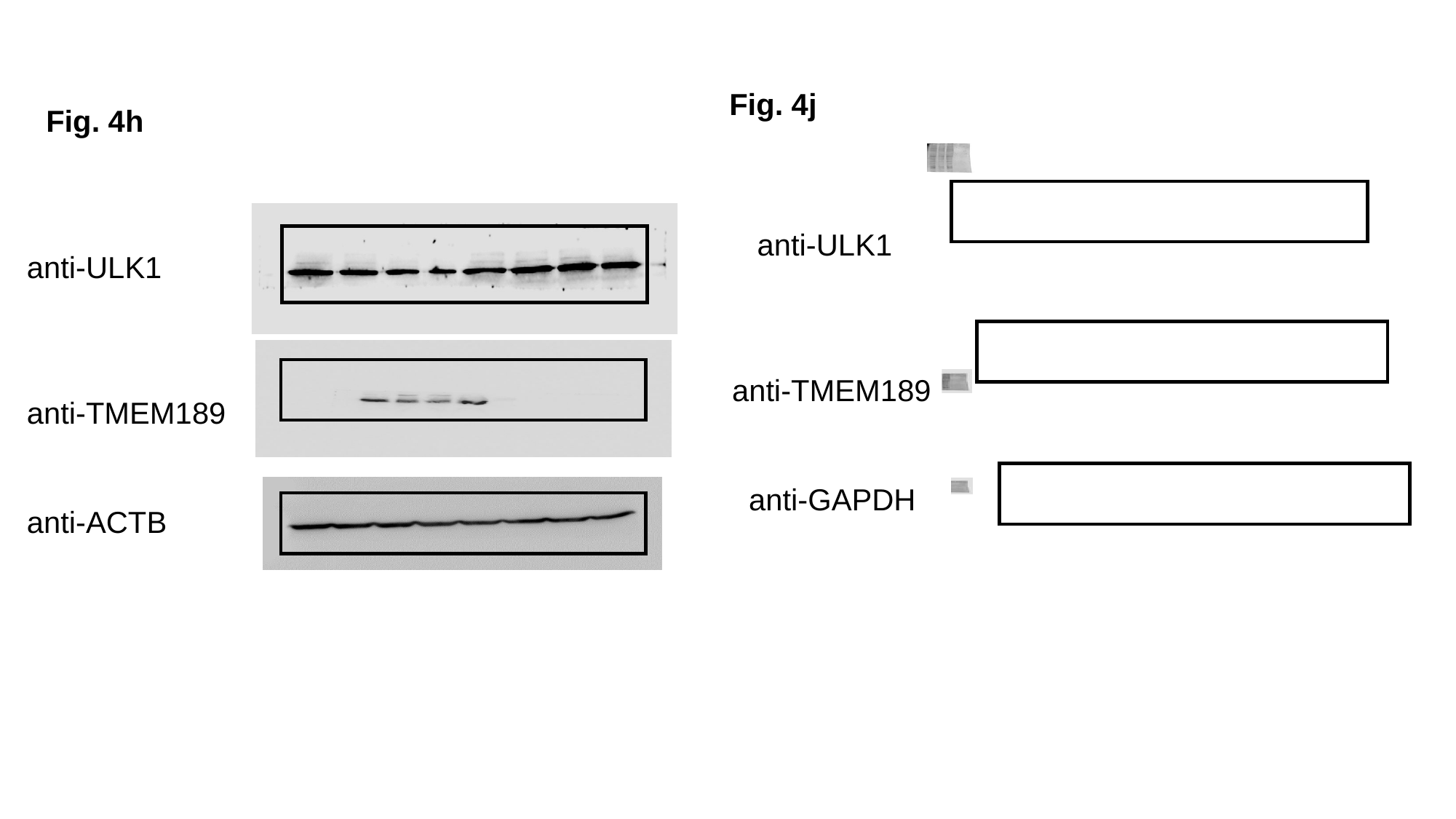

Fig. 4j
Fig. 4h
 anti-ULK1
 anti-TMEM189
 anti-GAPDH
anti-ULK1
anti-TMEM189
anti-ACTB

## Slide 12
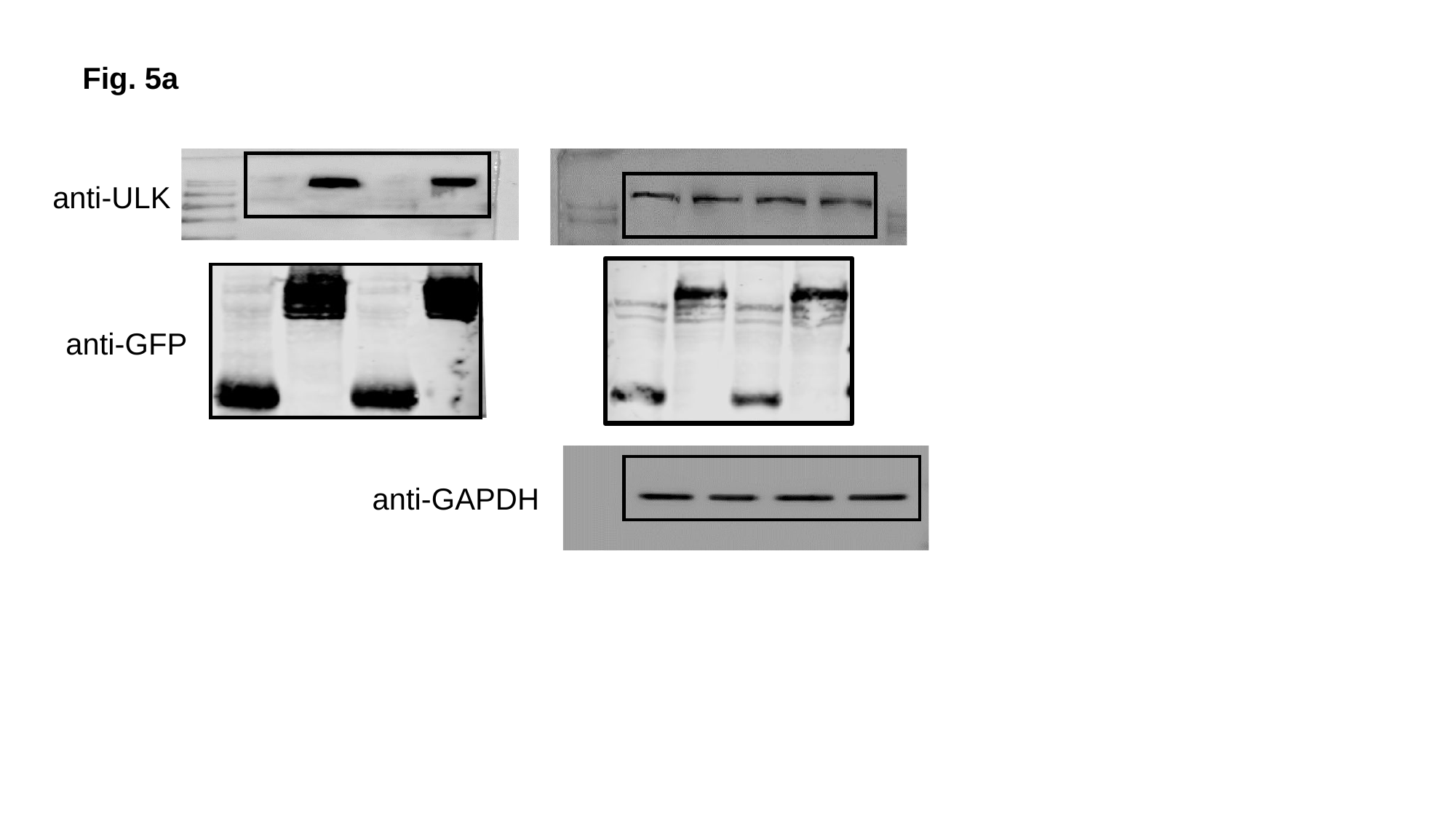

Fig. 5a
 anti-ULK
 anti-GFP
 anti-GAPDH

## Slide 13
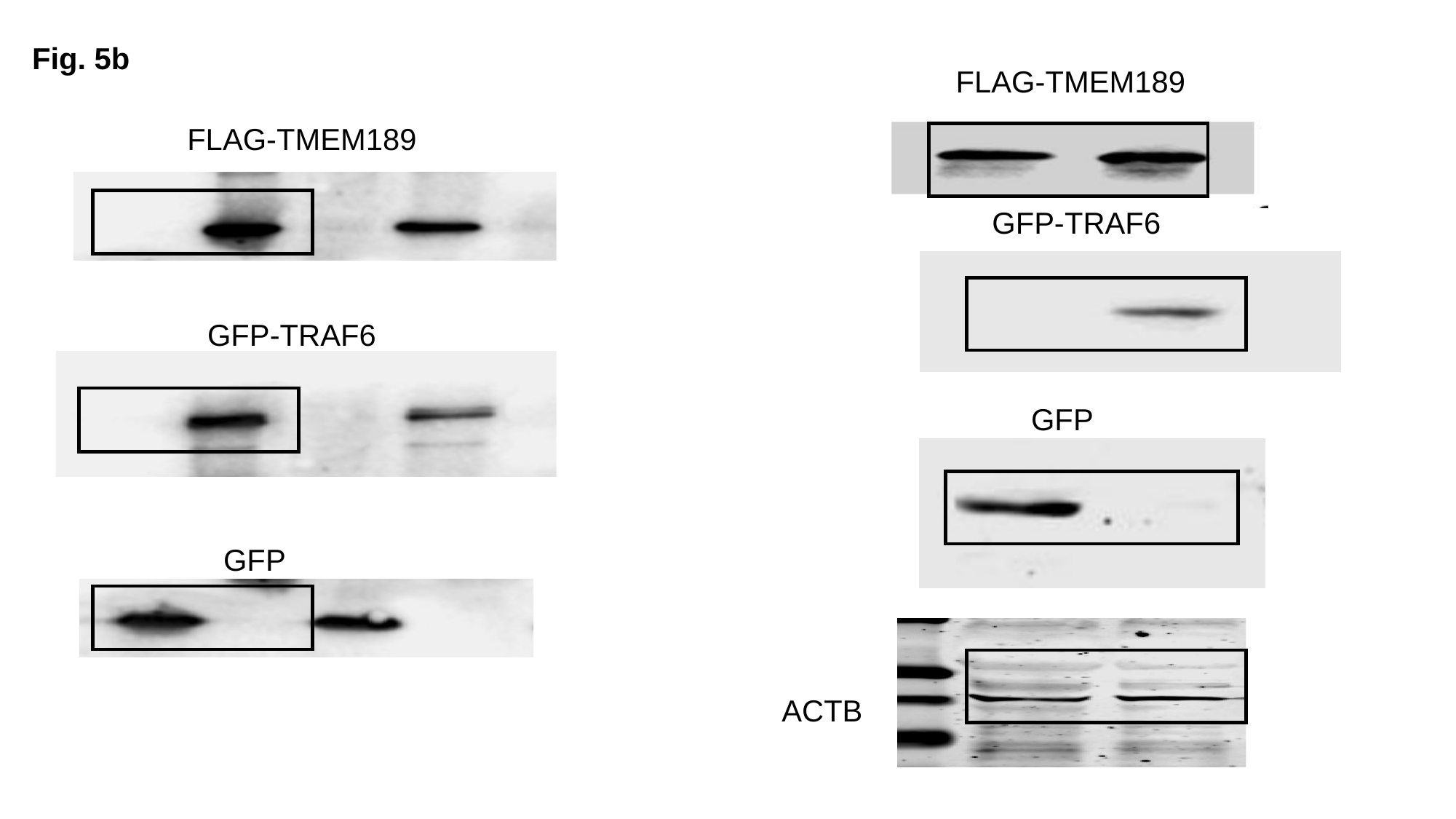

Fig. 5b
FLAG-TMEM189
FLAG-TMEM189
GFP-TRAF6
GFP-TRAF6
GFP
GFP
ACTB

## Slide 14
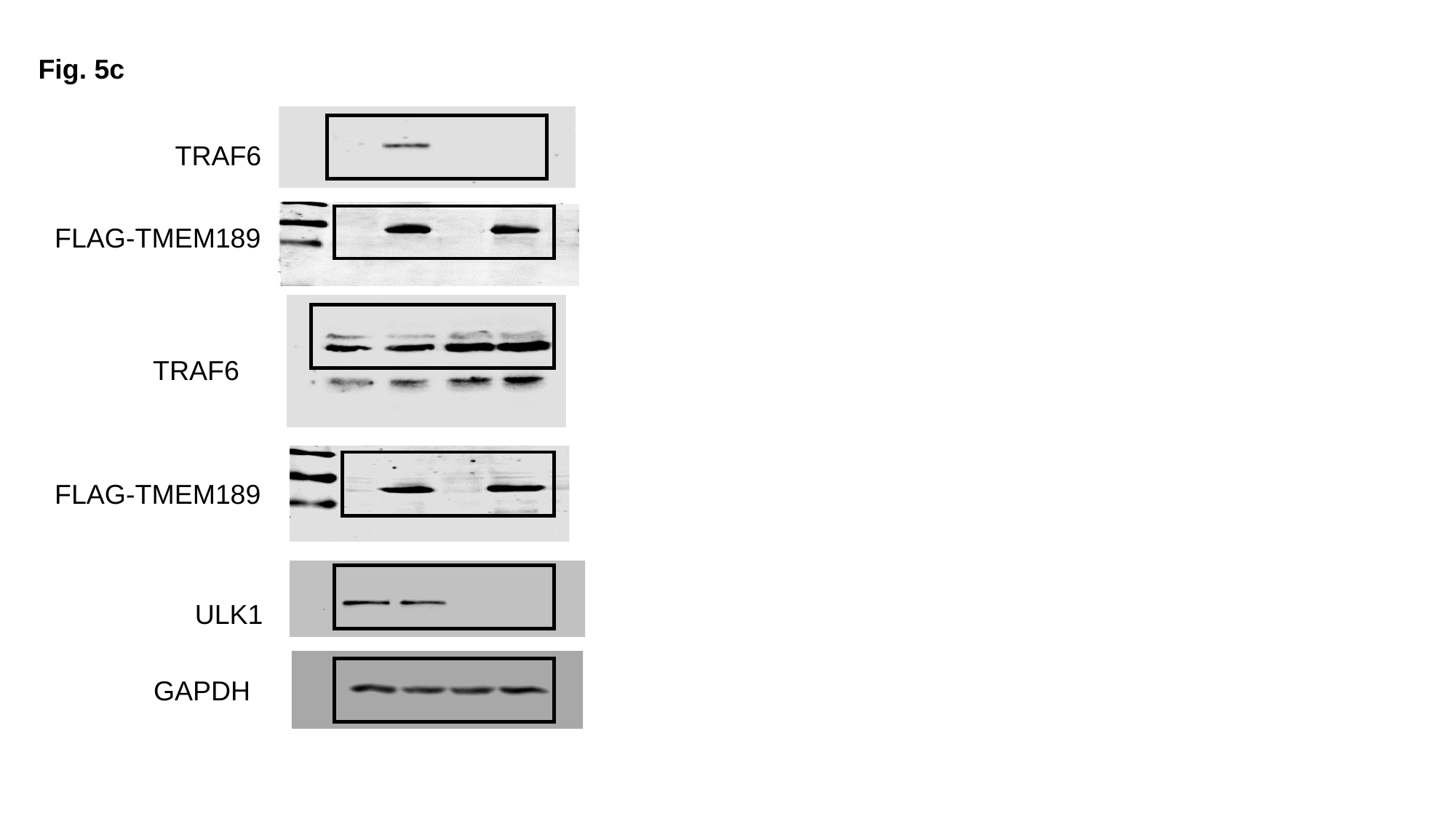

Fig. 5c
TRAF6
FLAG-TMEM189
TRAF6
FLAG-TMEM189
ULK1
GAPDH

## Slide 15
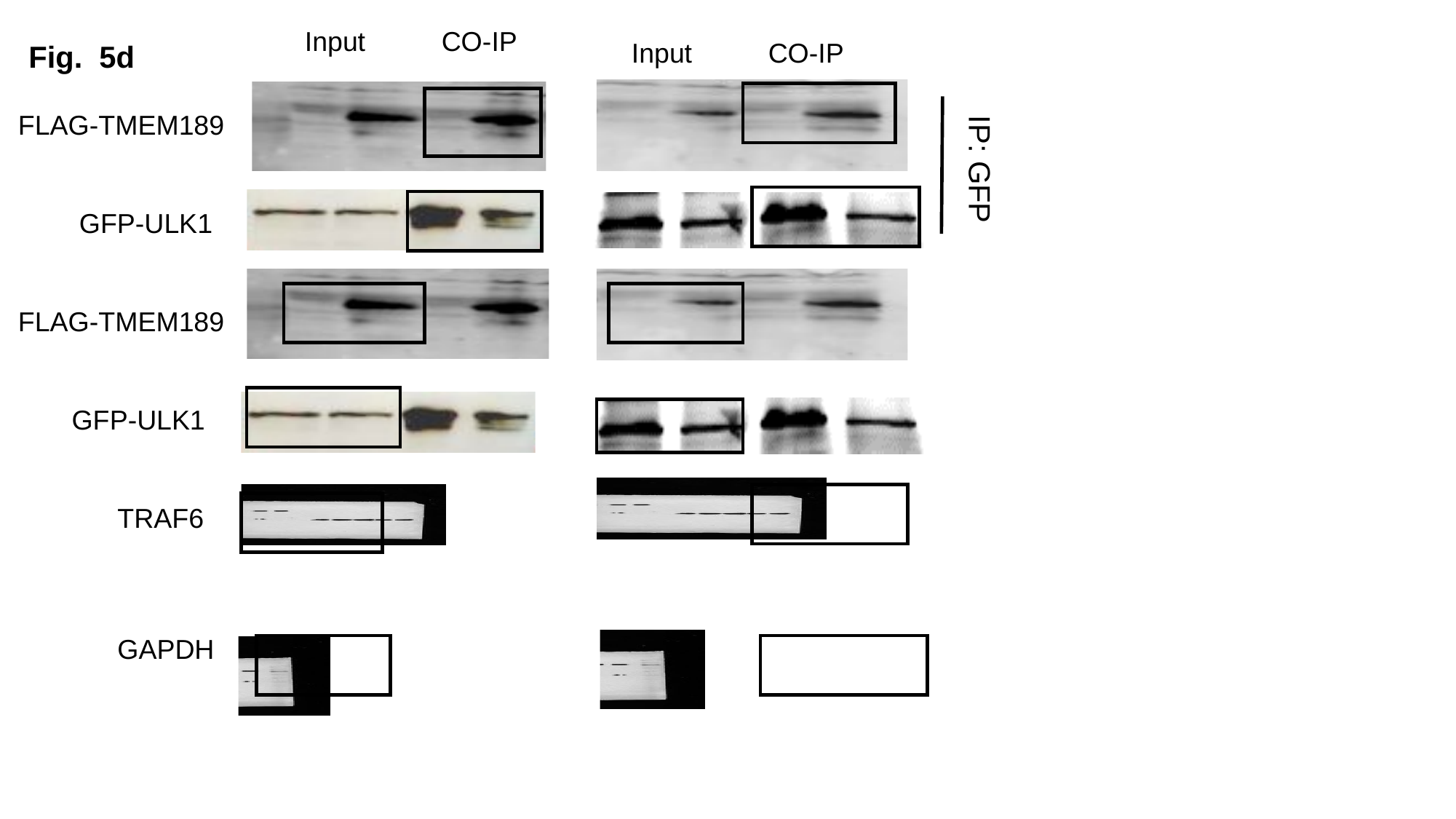

Input CO-IP
Input CO-IP
Fig. 5d
FLAG-TMEM189
 GFP-ULK1
FLAG-TMEM189
 GFP-ULK1
 TRAF6
 GAPDH
IP: GFP

## Slide 16
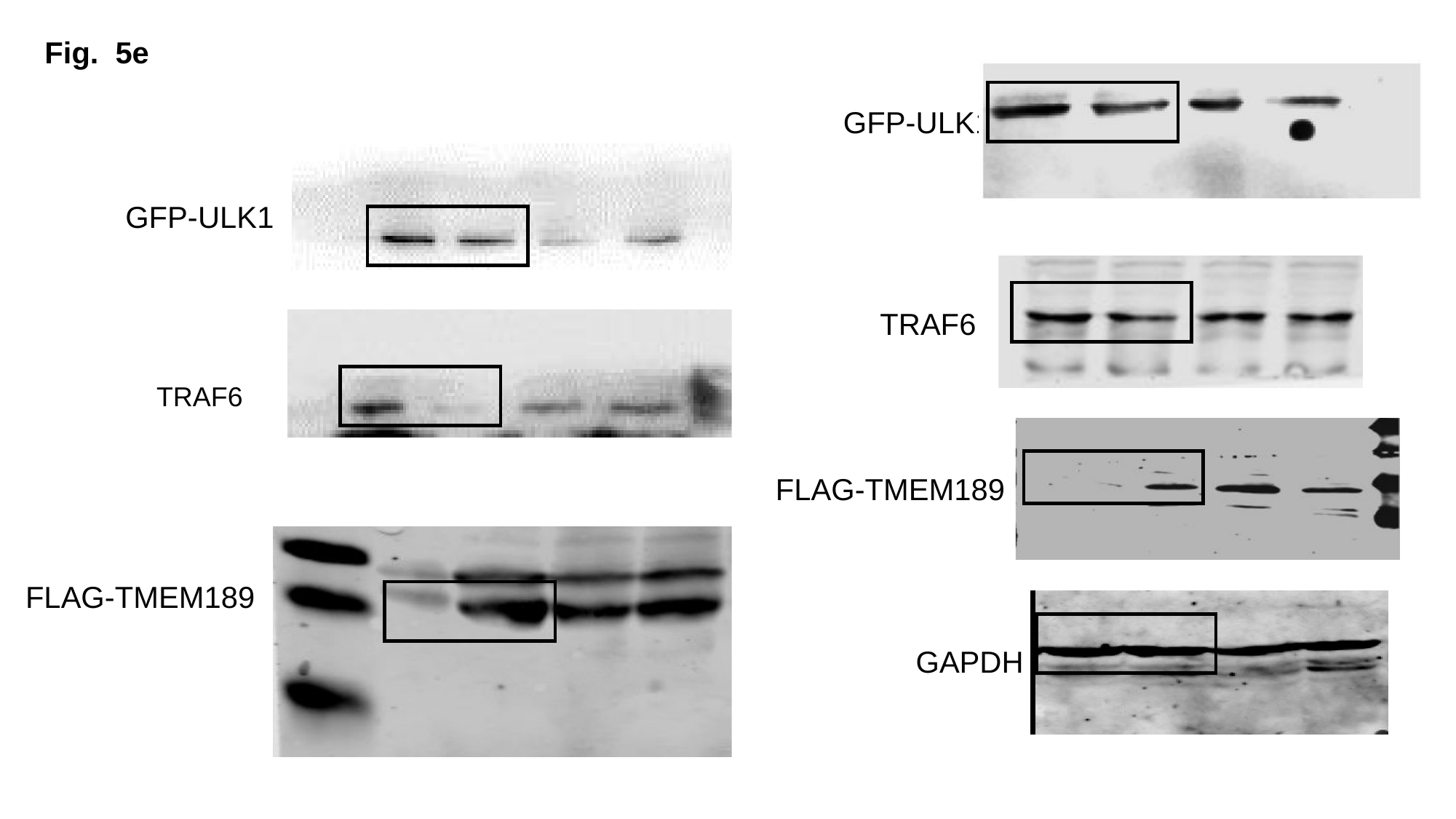

Fig. 5e
GFP-ULK1
GFP-ULK1
TRAF6
TRAF6
FLAG-TMEM189
FLAG-TMEM189
GAPDH

## Slide 17
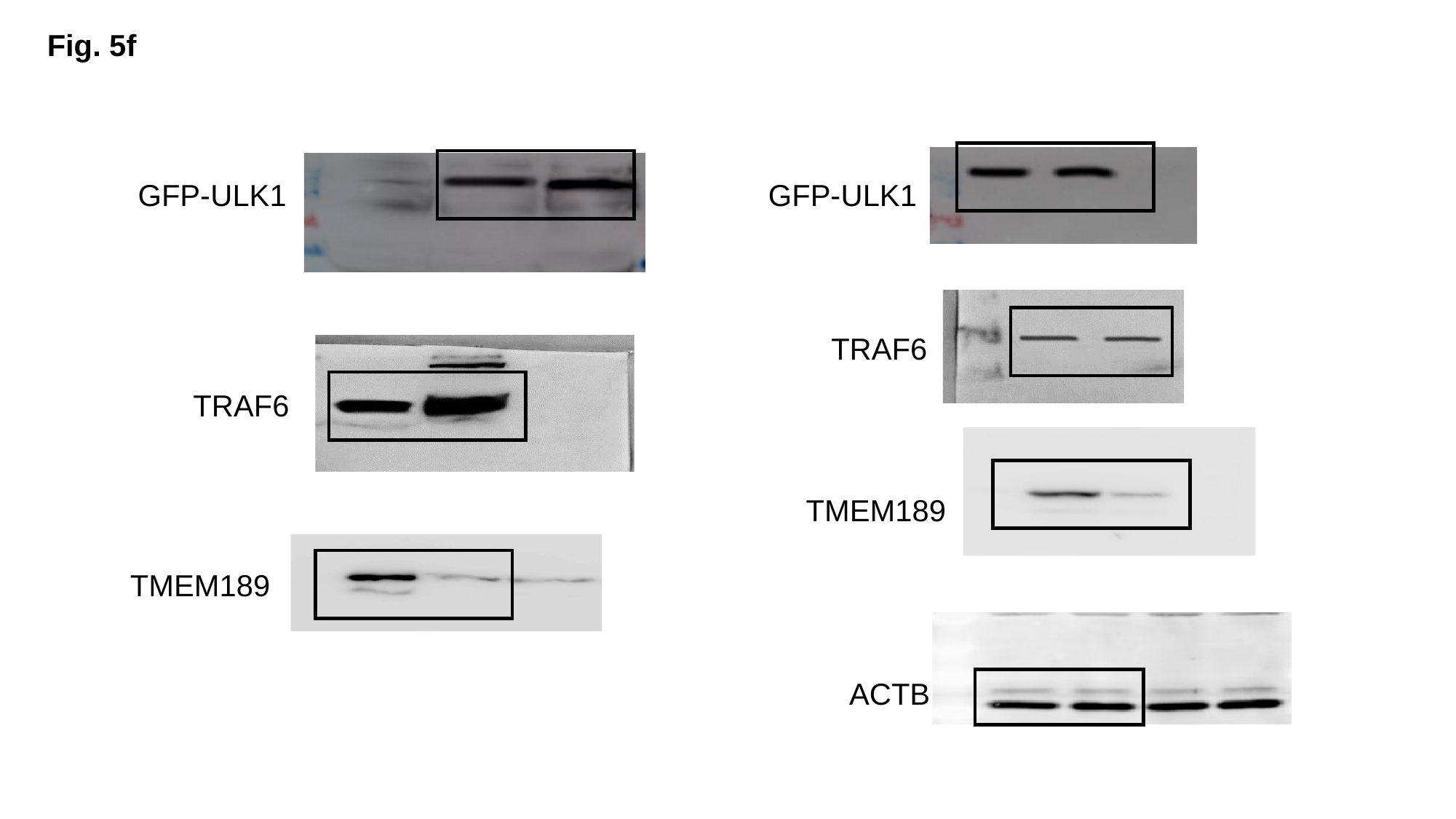

Fig. 5f
GFP-ULK1
GFP-ULK1
TRAF6
TRAF6
TMEM189
TMEM189
ACTB

## Slide 18
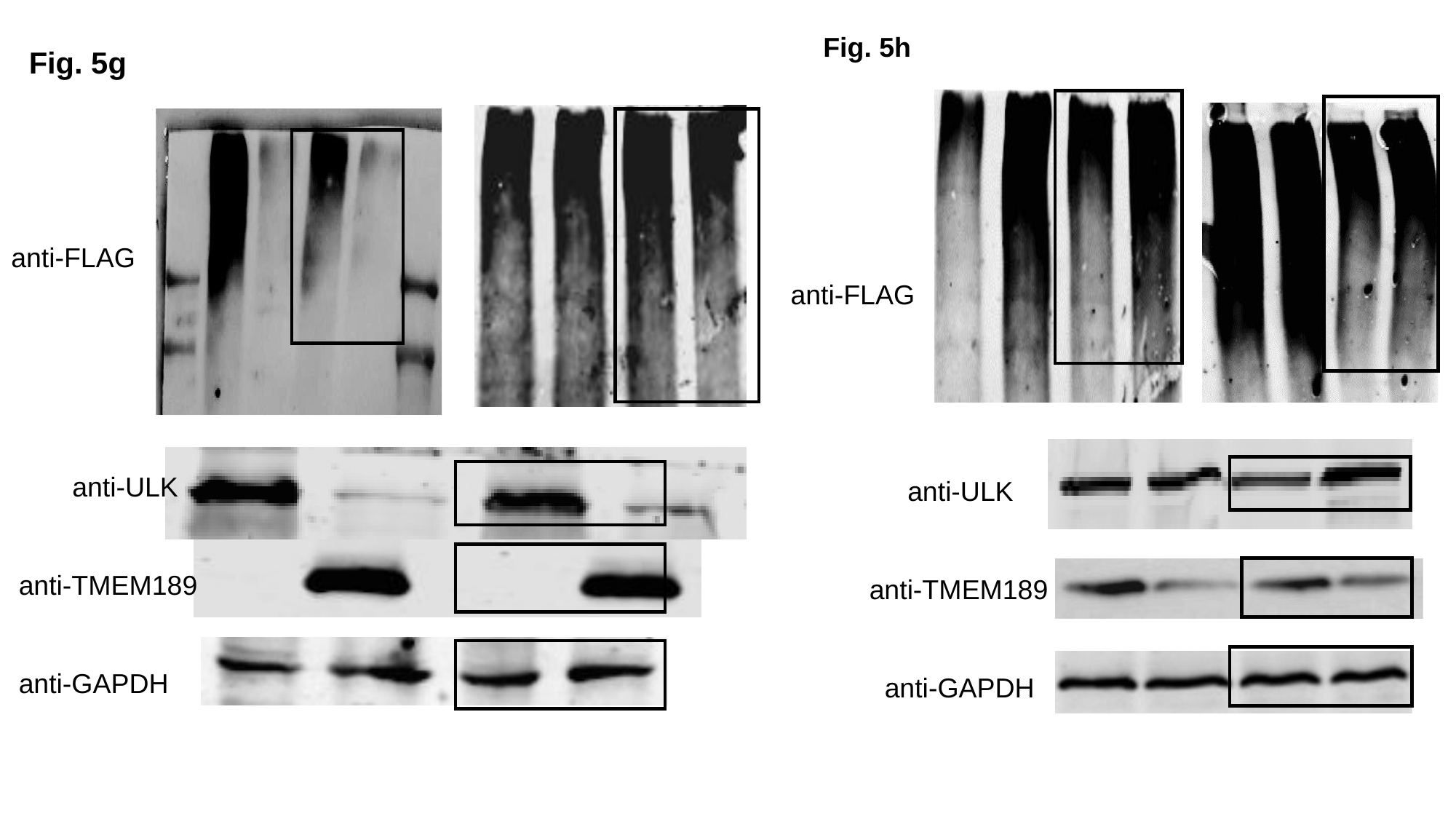

Fig. 5h
Fig. 5g
anti-FLAG
 anti-ULK
 anti-TMEM189
 anti-GAPDH
anti-FLAG
 anti-ULK
 anti-TMEM189
 anti-GAPDH

## Slide 19
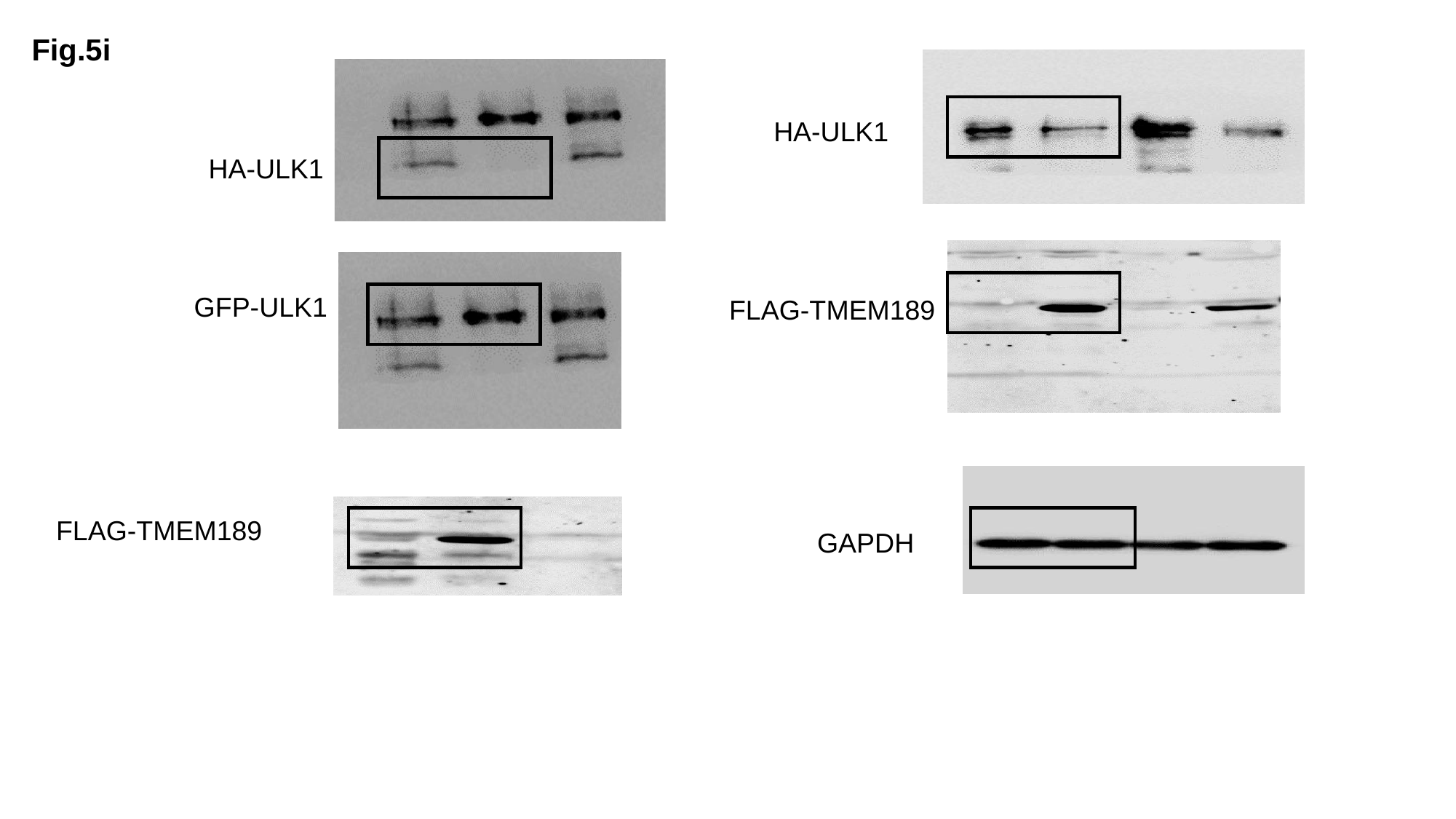

Fig.5i
HA-ULK1
HA-ULK1
GFP-ULK1
FLAG-TMEM189
FLAG-TMEM189
GAPDH

## Slide 20
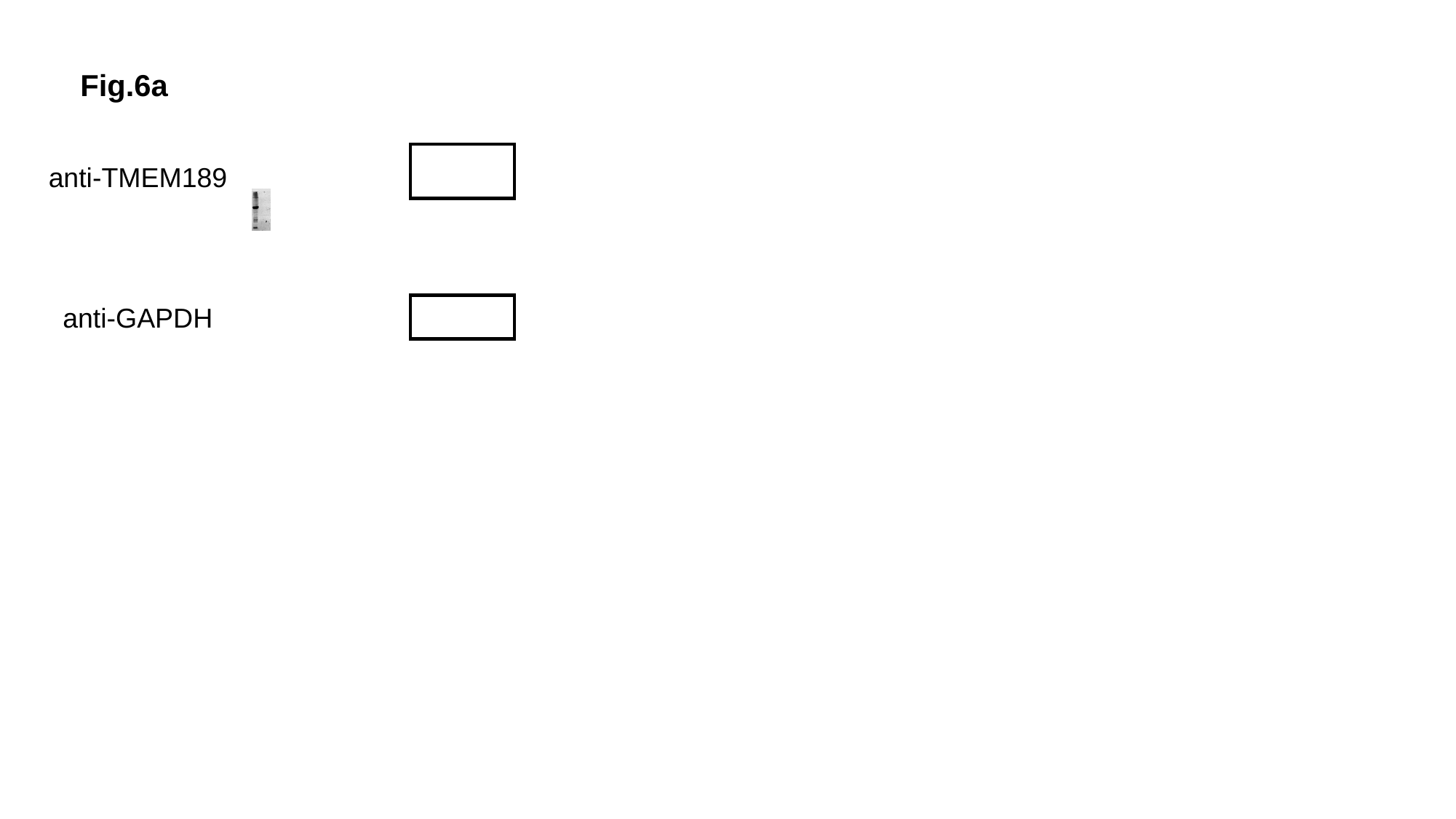

Fig.6a
anti-TMEM189
anti-GAPDH

## Slide 21
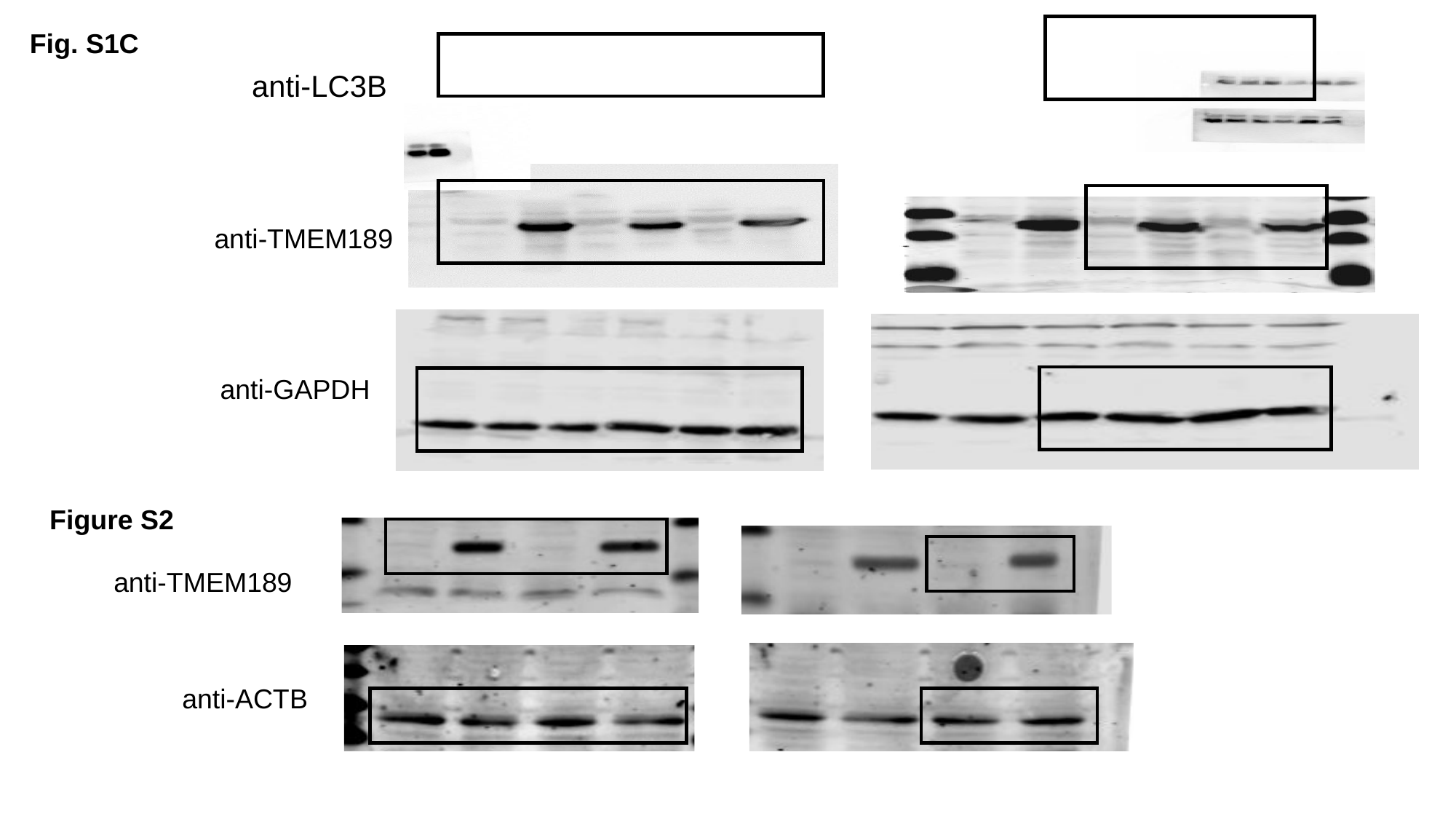

Fig. S1C
anti-LC3B
anti-TMEM189
anti-GAPDH
Figure S2
anti-TMEM189
anti-ACTB

## Slide 22
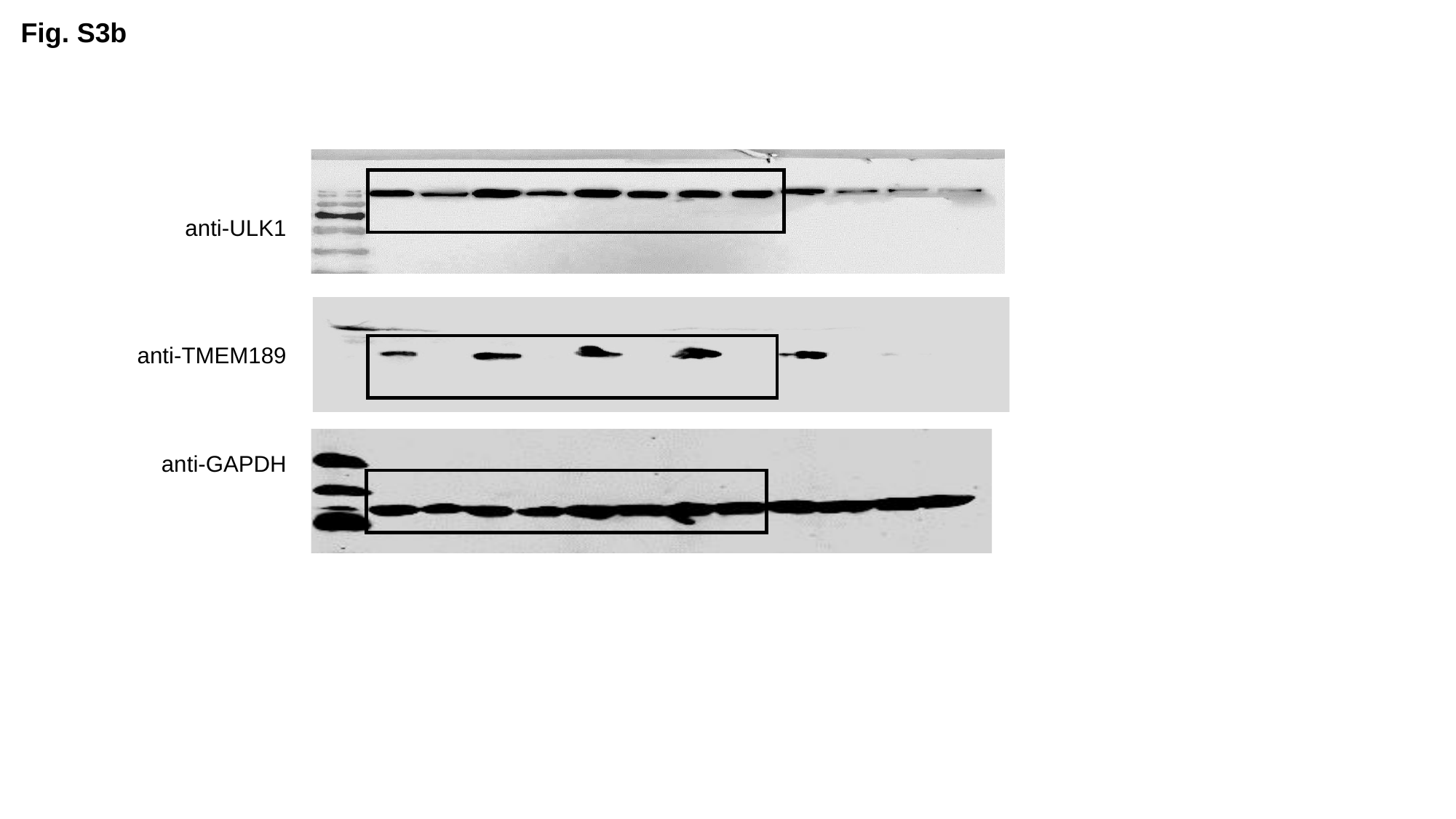

Fig. S3b
| anti-ULK1 |
| --- |
| anti-TMEM189 |
| anti-GAPDH |

## Slide 23
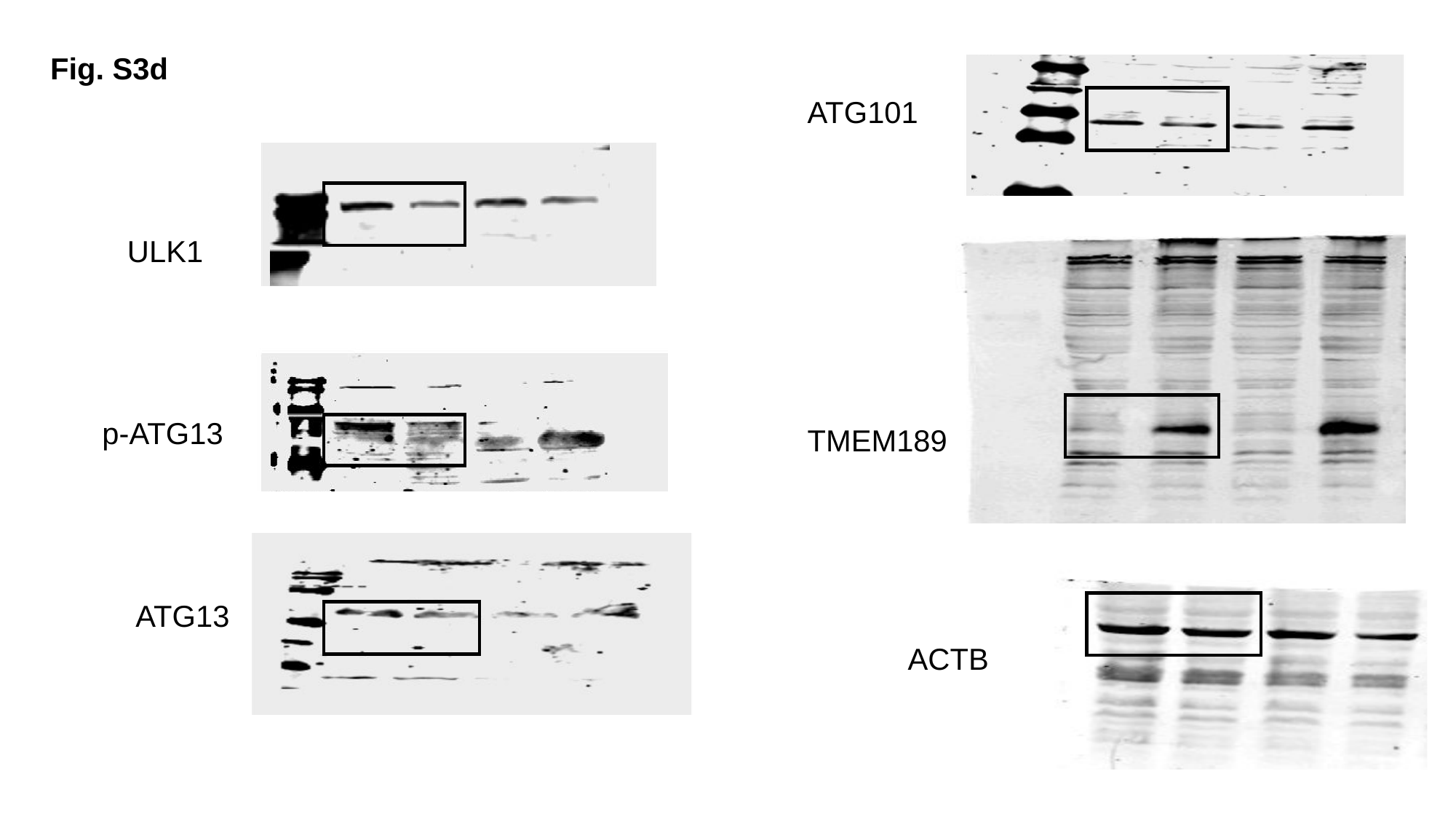

Fig. S3d
ATG101
TMEM189
 ACTB
 ULK1
p-ATG13
 ATG13

## Slide 24
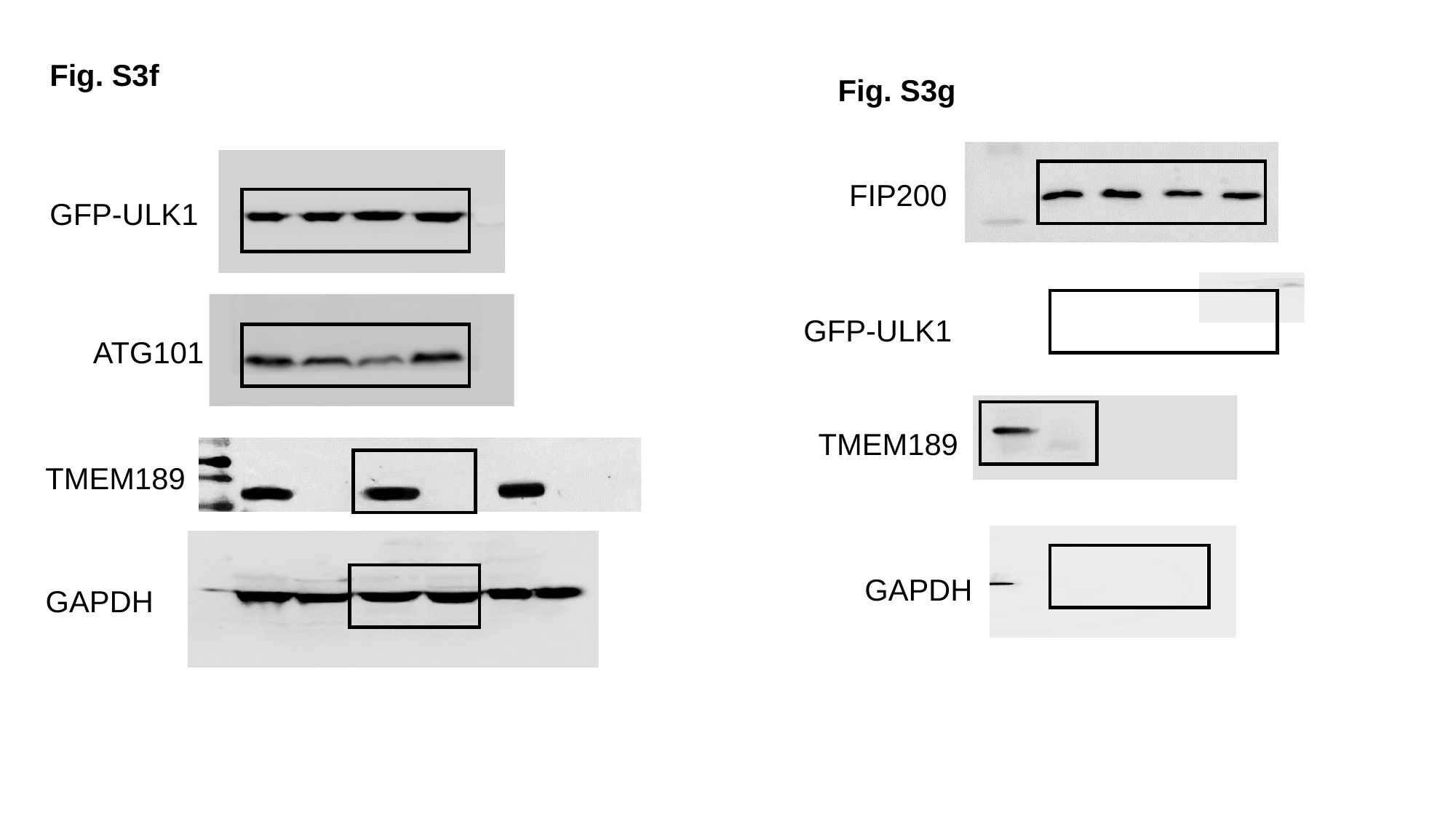

Fig. S3f
Fig. S3g
FIP200
GFP-ULK1
GFP-ULK1
ATG101
TMEM189
TMEM189
GAPDH
GAPDH

## Slide 25
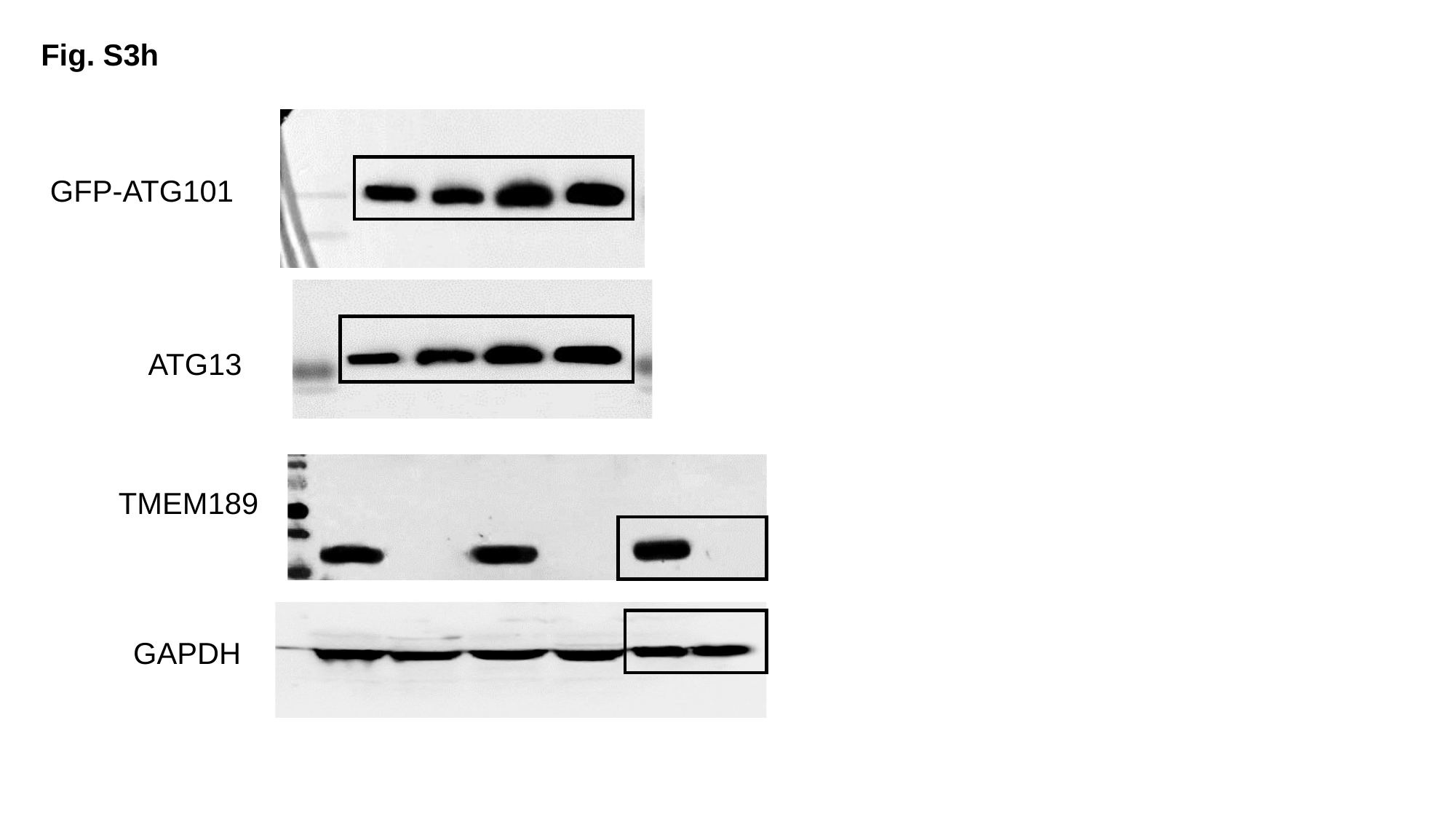

Fig. S3h
GFP-ATG101
ATG13
TMEM189
GAPDH

## Slide 26
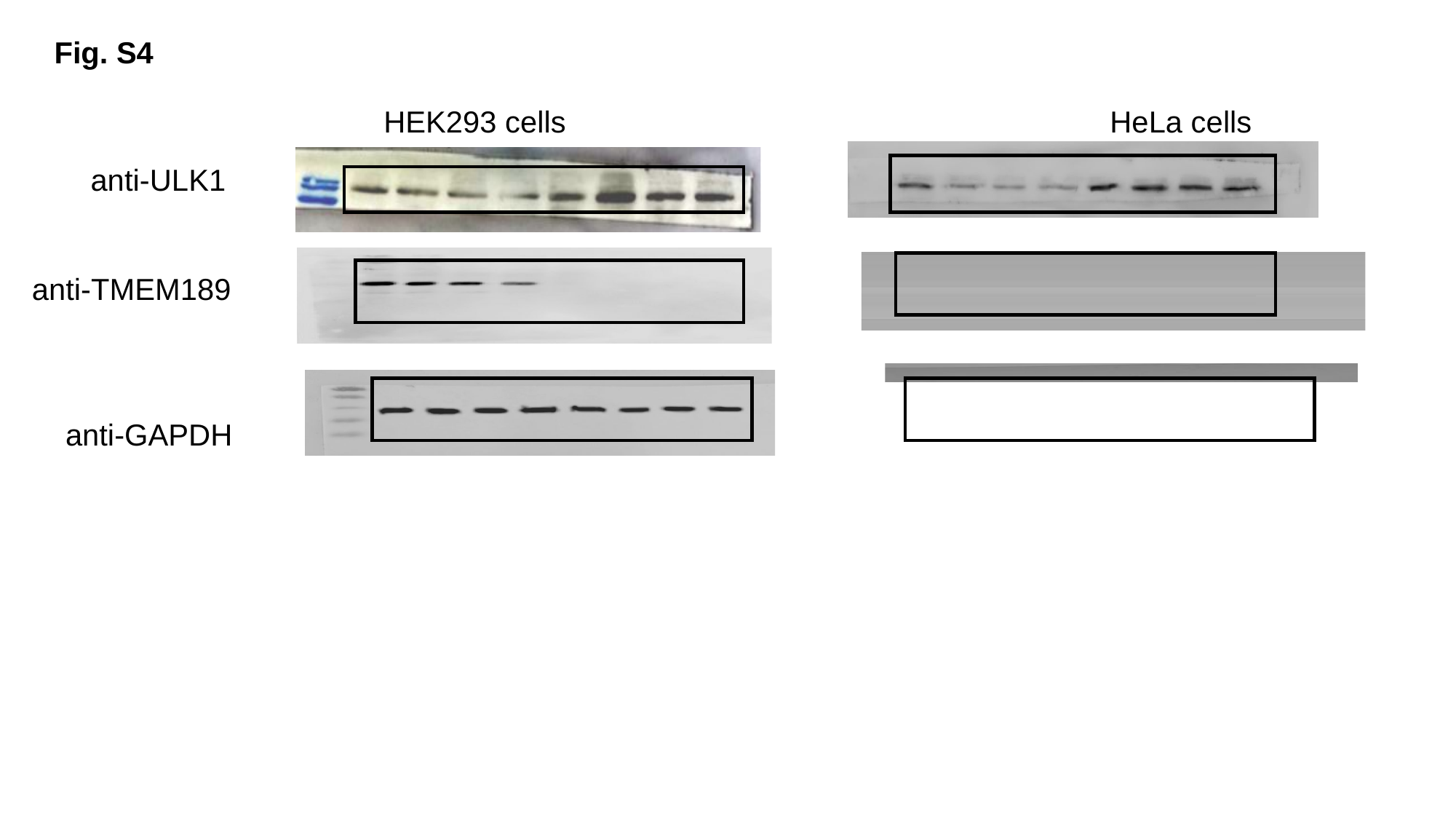

Fig. S4
HEK293 cells HeLa cells
 anti-ULK1
anti-TMEM189
 anti-GAPDH

## Slide 27
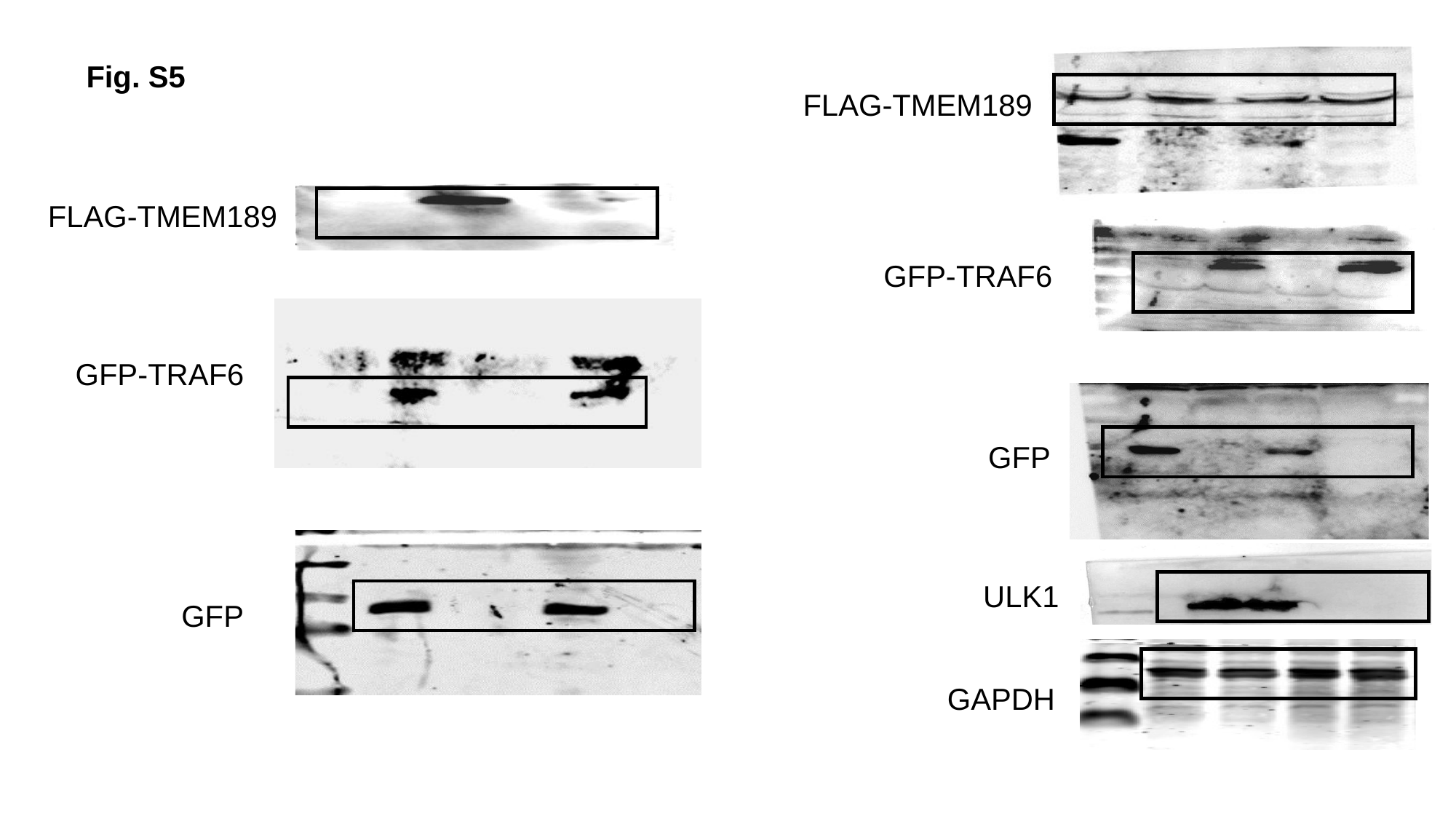

Fig. S5
FLAG-TMEM189
FLAG-TMEM189
GFP-TRAF6
GFP-TRAF6
GFP
ULK1
GFP
GAPDH

## Slide 28
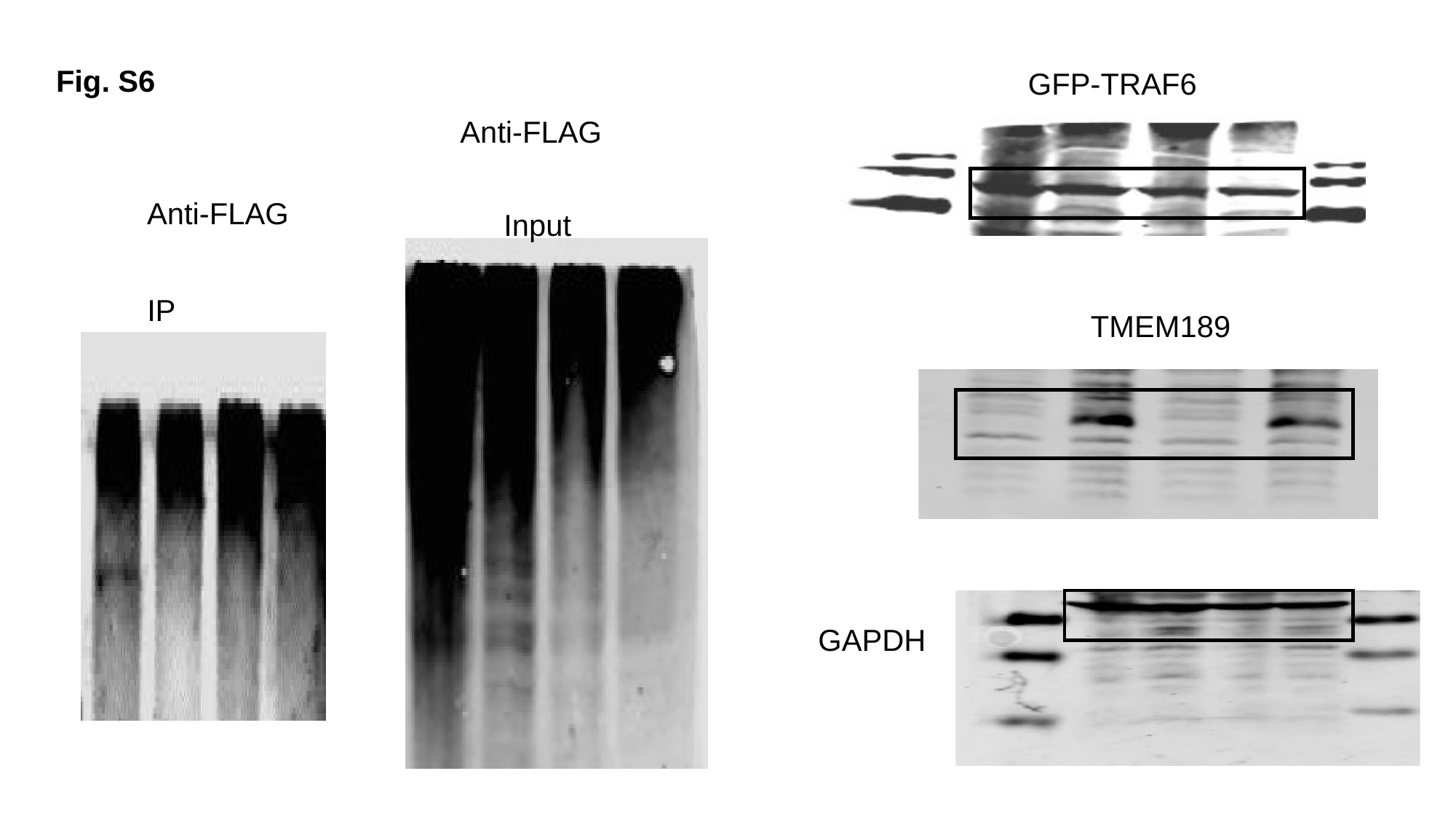

Fig. S6
GFP-TRAF6
Anti-FLAG
Anti-FLAG
Input
IP
TMEM189
GAPDH

## Slide 29
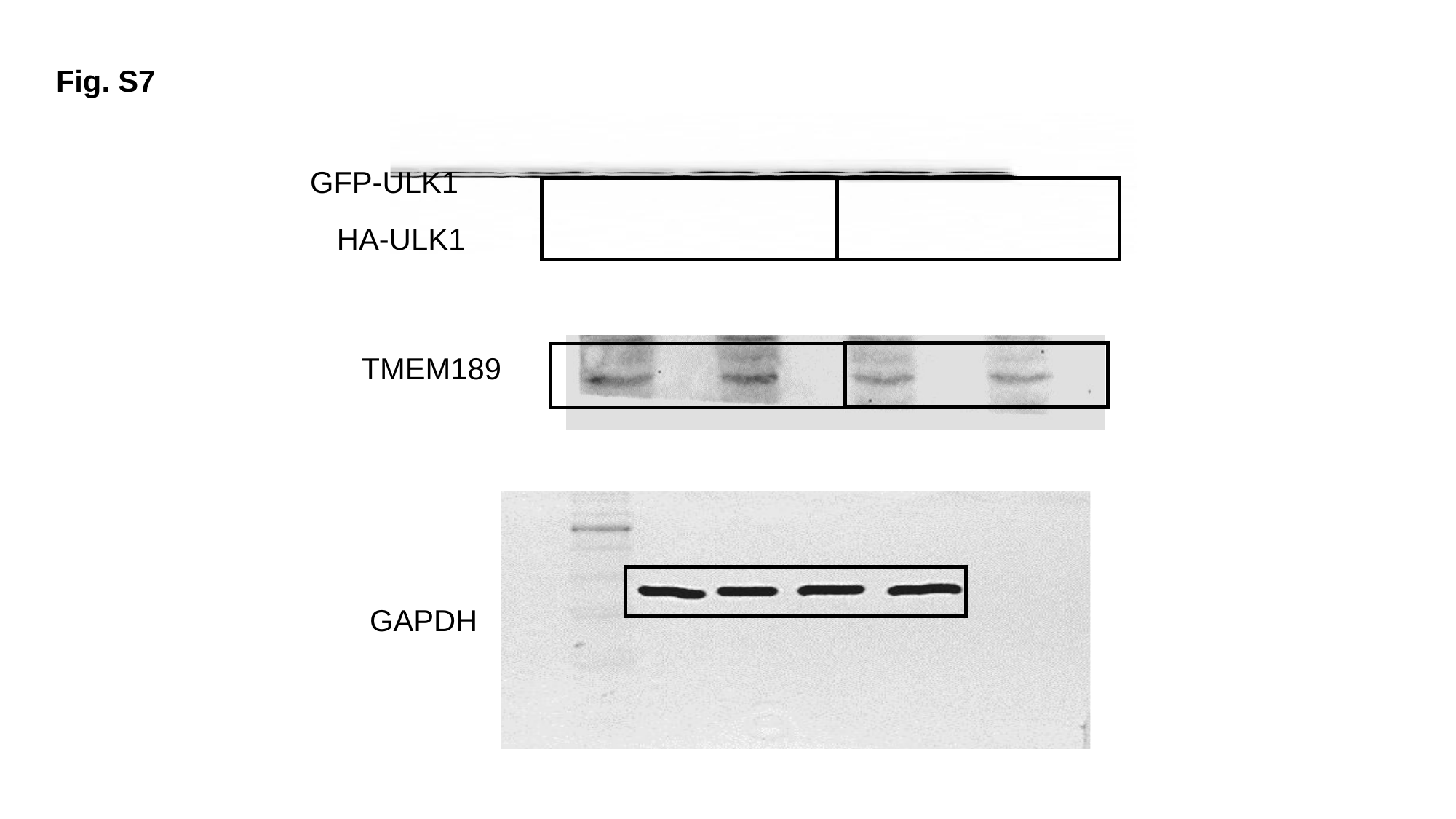

Fig. S7
GFP-ULK1
HA-ULK1
TMEM189
GAPDH
